# Supplementary material for: Impact of infectious diseases consultation on the management of Staphylococcus aureus bacteraemia in children
Source: BMJ Open. 2014 Jul 1;4(7):e004659. doi: 10.1136/bmjopen-2013-004659 (PMC4091395; doi:10.1136/bmjopen-2013-004659)
Supplement: Author's manuscript [file bmjopen-2013-004659.draft_revisions.pdf]

Impact of infectious diseases consultation on the management of S. aureus bactaeremia in children

Journal:	*BMJ Open*
Manuscript ID:	bmjopen-2013-004659
Article Type:	Research
Date Submitted by the Author:	10-Dec-2013
Complete List of Authors:	Saunderson, Rebecca; University of Cambridge, Department of Medicine<br>Gouliouris, Theodore; University of Cambridge, Department of Medicine<br>Cartwright, Edward; University of Cambridge, Department of Medicine<br>Nickerson, Emma; Cambridge University Hospitals NHS Foundation Trust, Department of Infectious Diseases<br>Aliyu, Sani; Cambridge University Hospitals NHS Foundation Trust, Department of Infectious Diseases<br>O'Donnell, Roddy; Cambridge University Hospitals NHS Foundation Trust, Department of Paediatrics<br>Kelsall, Wilf; Cambridge University Hospitals NHS Foundation Trust, Department of Paediatrics<br>Peacock, Sharon; University of Cambridge, Department of Medicine<br>Torok, Estee; University of Cambridge, Department of Medicine
**Primary Subject Heading**:	Infectious diseases
Secondary Subject Heading:	Epidemiology, Medical management
Keywords:	Epidemiology < INFECTIOUS DISEASES, Diagnostic microbiology < INFECTIOUS DISEASES, Cardiology < INTERNAL MEDICINE

SCHOLARONE™
Manuscripts

Title

Impact of infectious diseases consultation on the management of *S. aureus* bactaeremia in children

Authors

Rebecca B. Saunderson,[1] Theodore Gouliouris,[1,2,3] Edward J. Cartwright,[1,3] Emma J. Nickerson,[4] Sani H. Aliyu,[2,4] D. Roddy O'Donnell,[5] Wilf Kelsall,[5] Sharon J. Peacock,[1,2,3,6] M. Estée Török[1,2,3]

Affiliations

1. Department of Medicine, University of Cambridge, Cambridge, United Kingdom

2. Department of Microbiology, Cambridge University Hospitals NHS Foundation Trust, Cambridge, United Kingdom

3. Public Health England, Clinical Microbiology and Public Health Laboratory, Cambridge, United Kingdom

4. Department of Infectious Diseases, Cambridge University Hospitals NHS Foundation Trust, Cambridge, United Kingdom

5. Department of Paediatrics, Cambridge University Hospitals NHS Foundation Trust, Cambridge, United Kingdom

6. Wellcome Trust Sanger Institute, Hinxton, United Kingdom

Correspondence:

Dr M. Estée Török

University of Cambridge, Department of Medicine, Box 157, Addenbrooke's Hospital, Hills Road, Cambridge CB2 0QQ, United Kingdom

For peer review only

Telephone: +44 (0) 1223 336 845

Facsimile: +44 (0) 1223 336 846

Email: et317@medschl.cam.ac.uk

Key words

Staphylococcus aureus bacteraemia; infectious diseases consultation; management; outcome

Word count

Abstract = 260 words; Main text = 3214; Tables = 4; Figures=1

For peer review only

ABSTRACT

Objectives

Infectious diseases consultation (IDC) in adults with *Staphylococcus aureus* bacteraemia (SAB) has been shown to improve management and outcome. The aim of this study was to evaluate the impact of IDC on the management of SAB in children.

Study design

Observational cohort study of children with SAB

Setting

Cambridge University Hospitals NHS Foundation Trust, a large acute NHS Trust in the United Kingdom.

Participants

All children with SAB admitted to the Cambridge University Hospitals NHS Foundation Trust between 16 July 2006 and 31 December 2012.

Methods

Children with SAB between 2006 and 31 October 2009 were managed by routine clinical care (pre-IDC group) and data were collected retrospectively by case notes review. An IDC service for SAB was introduced in November 2009. All children with SAB were reviewed regularly and data were collected prospectively (IDC group) until 31 December 2012. Baseline characteristics, quality metrics, and outcome were compared between the pre-IDC group and IDC group.

Results

There were 66 episodes of SAB in 63 children; 28 patients (30 episodes) in the pre-IDC group, and 35 patients (36 episodes) in the IDC group. The median age was 3.4 years (IQR 0.2 – 10.7 years). Patients in the IDC group were more likely to have echocardiography performed, a removable focus of infection identified, and to receive

For peer review only

a longer course of intravenous antimicrobial therapy. There were no differences in total duration of antibiotic therapy, duration of hospital admission, or outcome at 30 or 90 days following onset of bacteraemia.

Conclusions

IDC resulted in improvements in the investigation and management of SAB in children.

For peer review only

ARTICLE SUMMARY

Strengths and limitations of the study

- This is the first study to examine the impact of introduction of infectious diseases consultation (IDC) on the management of *S. aureus* bacteraemia (SAB) in children.
- We found that IDC was associated with an improvement in investigation and management of SAB, but there was no difference in mortality between the pre-IDC and IDC groups.
- The main limitation of the study was the size of the study population, which may explain the lack of mortality benefit.
- The study was conducted in a tertiary referral centre, where clinical management is likely to have been good prior to introduction of the IDC, and may not be generalizable to other settings.

For peer review only

MAIN TEXT

Introduction

Staphylococcus aureus bacteraemia (SAB) is a serious infection that leads to significant morbidity and mortality in adults and children.[1,2] *Staphylococcus aureus* (*S. aureus*) causes significant disease in the paediatric population, occurring in 1.5% of all neonatal intensive care unit (ICU) admissions[3] and 6 per 100,000 children older than 1 year of age.[4] In neonates, SAB is almost always hospital-acquired, and is frequently due to intravascular catheter (IVC) associated infections.[3–6] The majority of non-neonatal cases of SAB are community-acquired; those that are hospital-acquired infections are usually IVC-associated.[7,8]

Identified risk factors for the development of SAB in the paediatric population include having a pre-existing medical condition, prolonged hospitalization, the presence of an IVC, and HIV infection.[1,3–6,9,10] Mortality from SAB in the adult population is about 30%.[11] Mortality rates in the paediatric population tend to be lower, but can be up to 15% in neonates and/or children with co-morbidities.[1,5,9,12,13] Given that SAB causes a substantial burden of disease in the paediatric population, strategies to improve management, prevent the complications of SAB, and reduce mortality are a clinical priority.

The impact of infectious disease consultation (IDC) in adults with SAB has been extensively.[14–24] IDC has been associated with improved adherence to evidence-based practice, including appropriate and targeted investigation, optimal duration of antibiotic therapy, and a reduction in complicated infection, morbidity and mortality.[14,16,17,22,23,25–28] In contrast, the impact of an IDC on the management and

For peer review only

outcomes of SAB in children has not previously been evaluated. The aim of this study was to determine the effects of routine IDC on the investigation, management, and outcome of children with SAB.

Materials and methods

Study setting and participants

Cambridge University Hospitals National Health Service Foundation Trust (CUH) is a tertiary referral centre for paediatrics in the East of England. The paediatric service has a 22-bed medical and surgical ward, a 17-bed paediatric haematology and oncology ward, an 11-bed paediatric ICU and high dependency unit (both caring for children aged from 0 to 16 years), and a 12-bed surgical and medicine ward for children aged up to 3 years. The Rosie Hospital, the on-site mother and baby hospital, has a 17-cot Neonatal ICU and a 10-cot Special Care Baby Unit.

Study design

We conducted an observational cohort study of all children with SAB admitted to CUH from 16 July 2006 until the 31 December 2012. In November 2009 an IDC service for all patients with SAB was established at CUH. IDC comprised an initial clinical review, followed by weekly follow-up until the time of hospital discharge. Data were collected from 2006 to 2009 by a retrospective review of the medical records, and prospectively thereafter during the IDC service. Demographic, clinical and microbiology information were collected using a case record form, and entered into an electronic database. Patients with contaminated blood cultures or polymicrobial blood cultures were excluded from the analysis.

For peer review only

Microbiological investigation

Blood cultures were collected and incubated at 37°C for 5 days using BacT/Alert 3D system (bioMérieux, Basingstoke, UK). Blood cultures that flagged positive were examined by microscopy and presumptively identified as *S. aureus* using a thermostable nuclease test.[29] Colonies of *S. aureus* were identified by routine methods after a further overnight incubation. Antibiotic susceptibilities were determined using disc diffusion testing, according to British Society for Antimicrobial Chemotherapy standards.[30] Throughout the study period a medical microbiologist provided telephone advice to the clinical team for all patients with SAB, and attended weekly ward rounds on the paediatric oncology ward and paediatric ICU.

Study procedures

From November 2009, all patients with SAB were reviewed by a member of the IDC team following presumptive identification of *S. aureus* in the blood culture. The assessment included clinical history to determine symptoms of infection, and physical examination to determine possible foci of infection.

An IVC was considered to be the focus of infection if there was evidence of inflammation at the catheter exit site and/or a vascular catheter tip culture positive for *S. aureus,* without clinical evidence of another source of bacteraemia.[31] Thrombophlebitis was diagnosed when there was clinical evidence of infection and inflammation along a blood vessel or when ultrasound or other imaging confirmed the presence of intravascular thrombosis in the setting of suspected infection. Bone and joint infections were defined according to United States Centers for Disease Control criteria.[32] The lung was considered to be the source of infection when there was

For peer review only

clinical, radiological and/or microbiological evidence of pulmonary infection. Soft tissue infection was considered to be the source of the bacteraemia if the clinical signs of a known or suspected soft tissue infection pre-dated or were present at the time of bacteremia.[14] A deep tissue abscess was defined by radiological imaging criteria. Infective endocarditis was diagnosed according to the modified Duke criteria.[33,34]

A SAB episode was defined as being greater than or equal to 14 days from a previous episode, in the absence of persistent bacteraemia or focus of infection. A secondary site of infection was defined as a site of infection separate from the primary site of infection that was not present at the time of the initial examination. Healthcare-associated bloodstream infection was defined according to previously published criteria.[35] Hospital-acquired infection was defined according to United States Centers for Disease Control.[32] Community-acquired infections were defined as those patients with a positive blood culture taken at or within 48-hours of admission who did not meet criteria for healthcare-associated bloodstream infection.[35] Patients were classified as having uncomplicated SAB if blood cultures were negative two to four days after the initial blood culture was positive, if they had defervesced at 72 hours, if there was no evidence of metastatic disease or endocarditis, or if they had a catheter related infection.[36]

Appropriate antimicrobial therapy was defined as therapy to which the isolate was determined to be susceptible by antimicrobial disc susceptibility testing. The duration of therapy was the length of time that a patient received antibiotics to which the isolate was susceptible. An underlying medical condition was defined as any chronic medical condition that was present at the time of bacteraemia. C-reactive protein,

For peer review only

white cell counts, and platelets were measured on the day of or within 48 hours post bacteraemia. Duration of hospital admission and outcome at 30 and 90 days post-bacteraemia were recorded for all patients.

Treatment recommendations

Antimicrobial treatment recommendations were provided for all children with SAB, based on existing evidence on the management of SAB in adults.[14,37–40] These included removal of a removable focus of infection,[14] performing repeat blood cultures at 48 to 96 hours,[37] performing a transthoracic echocardiogram, performing radiological imaging of suspected deep foci of infection, treating uncomplicated infection with 14 days of intravenous (IV) antibiotics,[38] treating complicated infections with a minimum of 28 days of IV antibiotics[39] and using beta-lactam therapy as the mainstay of treatment for methicillin-susceptible *S. aureus*.[40]

Statistical analysis

Data were analyzed using STATA version 12 (StataCorp, College Station, Texas, USA). Categorical variables were analyzed using the Fisher's exact test and reported as the number and percent. Continuous variables were compared using the Mann Whitney U test and reported as the median and interquartile range.

Ethics statement

Written informed consent from participants was not required as the study was conducted as a service evaluation. The study was approved by the University of Cambridge Human Biology Research Ethics Committee and the CUH Research and Development Department.

For peer review only

Results

Patient characteristics

Between July 2006 to December 2012, 71 children had one or more blood cultures that were positive for *S. aureus.* Sixty-three children (66 episodes) were included in the study. Five children (six episodes) were excluded because of polymicrobial infection. Three patients (three episodes) were excluded when the culture was interpreted as representing contamination following clinical assessment. Thus, 28 patients (30 episodes) were included in the pre-intervention (pre-IDC) group, and 35 patients (36 episodes) in the intervention (IDC) group. The study schema is summarized in Figure 1. Four patients (14.3%) received an IDC before the service was implemented in 2009 and 34 patients (94.4%) received an IDC after the service was implemented in 2009.

The baseline characteristics of the study population are presented in Table 1. These were similar apart from a higher serum C-reactive protein in the IDC group (448 nmol/L versus 333nmol/L, P=0.047). The clinical features for SAB were likewise similar, apart from an increased proportion of IVC-associated infections in the IDC group (61.3% versus 26.7%, P=0.013) (Table 2). A higher proportion of patients had an unidentified focus of infection in the pre-IDC group compared with the IDC group (23.3% versus 5.6%, P=0.068). The risk factors for SAB were also similar apart from an increased frequency of IVC in the IDC group (63.9% versus 43.3%, P=0.137) (Table 3).

Clinical management

For peer review only

A service evaluation of the IDC service was conducted, the results of which are summarized in Table 4. In the IDC group, 34/36 episodes had an infectious diseases review with a median time to review of two days (range 1 to 4 days). Patients in the IDC group were more likely to have a transthoracic echocardiogram performed (80.6% versus 33.3%, P=0.0001). They were also more likely to have a removable focus of infection identified (43.9% versus 23.3%, P=0.003), although there was no difference between the two groups in the likelihood of removal, or the time to removal. There was no difference in the number of repeat blood cultures performed between groups.

Antimicrobial therapy

There was no difference between the two groups in the time taken to initiate appropriate antimicrobial therapy. Patients in the IDC group were more likely to receive a longer duration of IV antimicrobial therapy (18 days versus 13.5 days, P=0.035), although there was no difference in total duration of therapy (IV and oral) between the two groups. In patients with complicated SAB, the duration of IV antibiotic therapy was longer in the IDC group (22 days versus 14 days, P=0.02) although there was no difference in total duration of antibiotic therapy (IV and oral) between the two groups. Patients in the IDC group were more likely to receive a longer duration of IV therapy if their repeat blood culture result was positive (P=0.007). In patients with uncomplicated SAB there was no difference between groups in the duration of IV antibiotics, or the total duration of antibiotic therapy. In terms of compliance with recommended standards for duration of therapy, patients in the IDC group were more likely to meet these standards compared with patients in the non-IDC group, both for complicated SAB (42.1% versus 13.3%, P=0.13) and

For peer review only

uncomplicated SAB (68.4% versus 46.7%, P=0.14). There were no differences in the proportion of patients receiving beta-lactam therapy.

Outcome of SAB

The duration of hospital admission was similar in the pre-IDC and IDC groups, and in those with uncomplicated and complicated SAB (Table 4). SAB was recorded in the discharge summary in the majority of patients in both groups. Four secondary infections were diagnosed, three in the IDC group and one in the pre-IDC group. In the IDC group, the secondary infections were tricuspid valve endocarditis in a very low birth weight neonate with patent ductus arteriosus, pneumonia, and osteomyelitis, respectively. One child in the non-IDC group developed osteomyelitis. There were three cases of recurrent bacteraemia within 90 days, of which two were in the pre-IDC group and one was in the IDC group. Three children died within 30 days of SAB, all in the IDC group, giving an overall 30-day mortality rate of 4.8%. One death occurred in a child with metastatic cancer and was not attributed to the SAB. The other two deaths were deemed attributable to SAB, as blood cultures were positive at the time of death. One patient was a neonate and died prior to IDC, and the second case had cerebellar atrophy, developmental delay and was receiving total parenteral nutrition. The 90-day overall mortality rate was 7.9%. Two patients died between 30 and 90 days post-SAB, both in the pre-IDC group. One patient had metastatic cancer, and the other had complex congenital heart disease.

Discussion

To our knowledge this is the first study to systematically examine the impact of introduction of an IDC service on the management of SAB in children. We compared

For peer review only

the clinical features, management, and outcomes of all children with presenting with SAB to our hospital between July 2006 and December 2012, before and after introduction of an IDC service. The main findings of the study were that patients in the IDC group were more likely to have echocardiography performed, a removable focus of infection identified, and to receive a longer course of IV antibiotic therapy. These findings concur with those from previous studies of IDC conducted in adults with SAB, and reflect current best practice.

Follow-up blood cultures have been recommended in adults as prolonged bacteraemia is a predictor of complicated infection and poorer outcome in SAB.[37] As a result, prolonged IV antibiotic therapy is recommended in patients with positive repeat blood cultures. We found that children who had a positive repeat blood culture were more likely to receive a longer course of IV antibiotics if they were in the IDC group.

Echocardiography was performed in a higher proportion of children in the IDC group compared with the pre-IDC group. The rates of IE in children with SAB are reported to be between 0-20%, which is similar to rates reported in the adult population.[1,3,6,8,12,41–43] An American study by Valente and colleagues diagnosed IE in 20% of children with SAB (~12% of whom had confirmed IE).[41] Children with underlying congenital heart disease had a higher prevalence of confirmed or probable IE compared to those who had structurally normal hearts (53% versus 3%) and patients with definite IE had multiple positive blood cultures. Mortality was higher in patients with endocarditis compared to those without (40% versus 12%). Another study from South Africa reported an IE rate of 11% in children with SAB.[12] Risk factors for the development of IE in children include congenital heart disease, a

For peer review only

central IVC, and persistently positive blood cultures after 24 hours. [41,42] In the United Kingdom, there are no published guidelines on the use of echocardiography in children with SAB. The Infectious Diseases Society of America guidelines for MRSA bacteraemia recommend performing echocardiography in children with congenital heart disease, those with bacteraemia duration greater than two days, or those with other clinical findings suggestive of endocarditis.[36] In this study the one child who developed tricuspid valve endocarditis was a very low birth weight premature neonate with a patent ductus arteriosus, an IVC infection, and persistent bacteremia. Our findings concur with these guidelines, and support the use of echocardiography in children with SAB.

We also found that a higher proportion of children had an IVC-related infection, and/or a removable focus of infection in the IDC group compared with the pre-IDC group. Conversely, there were fewer patients with an unidentified focus of infection in the IDC group compared with the pre-IDC group. These findings indicate that the introduction of specialist infectious diseases review improved the rate of diagnosis of the focus of infection in children with SAB, suggesting that the consult service was beneficial.

In terms of duration of antimicrobial therapy, we found that patients in the IDC group received longer courses of IV antibiotics in complicated infection, compared with patients in the pre-IDC group. This corresponds with findings from studies in adults with SAB, and suggests that specialist infectious diseases review may be beneficial. However, no differences in morbidity or mortality between the pre-IDC and IDC groups were found. The most likely explanation for this was the small study

For peer review only

population (63 patients in total and only 35 in the IDC group). The mortality rate of SAB in children is low so a large number of patients would be required to demonstrate even in small difference in mortality. Furthermore the duration of antibiotic therapy was similar in the two groups, although the IDC group did receive longer courses of IV antibiotics. It may be that total duration of antibiotic therapy is more important than the route of administration provided that adequate concentrations can be achieved in the blood. Indeed studies to examine this question, comparing short versus long courses of IV antibiotics in *S. aureus* bacteraemia in adults are ongoing, and should also be conducted in the paediatric population. Finally, although a removable focus of infection was identified more frequently in the IDC group, the likelihood or removal and the time to removal did not differ; this may also explain the lack of difference in outcome between the two groups.

We acknowledge several limitations to our study. The study population was small and the differences in diagnosis and management that we observed in the two groups did translate into differences in outcome, as discussed above. The retrospective data collection during the pre-IDC period (2006 to 2009) carries a risk of incomplete recording of data and potential bias. There were, however, no differences in baseline characteristics between the two groups in terms of age, gender, underlying co-morbidities or focus of infection. The only exception was a higher frequency of IVC-related infections in the IDC group, which may have been under-reported in the pre-IDC period and/or diagnosed more frequently in the IDC period.

In conclusion, we found that introduction of IDC for paediatric SAB resulted in improvements in management and a more consistent approach to care across the

For peer review only

paediatric service. These findings concur with those of previous studies of IDC in adults with SAB and support the use of echocardiography in SAB in children, particularly for patients with risk factors for complicated disease. Despite improvements in the investigation and clinical management, we did not find any differences in the development of secondary infections, recurrent bacteremia, or death between the two groups. The most likely explanation for this is the small study population and larger prospective studies are required validate our findings and to determine the optimal strategies for investigation and management of paediatric SAB.

Funding

This work was supported by grants from the United Kingdom Clinical Research Collaboration (UKCRC) Translational Infection Research Initiative (TIRI); the Medical Research Council (G1000803), with contributions from the Biotechnology and Biological Sciences Research Council, the National Institute for Health Research (NIHR) on behalf of the United Kingdom Department of Health, and the Chief Scientist of the Scottish Government Health Directorate; the Public Health England; and the NIHR Cambridge Biomedical Research Centre.

Competing interests statement

All authors have no conflicts of interest to declare.

Author contributions

RBS was part of the infectious diseases consultation service, collected the data, performed the data analysis, and wrote the first draft of the manuscript. TG was part of the infectious diseases consultation service, collected the data, and contributed to

For peer review only

writing the manuscript. EJC collected the data, and contributed to the data analysis writing the manuscript. EJN was part of the infectious diseases consultation service, collected the data, and contributed to writing the manuscript. SHA was part of the infectious diseases consultation service, collected the data, and contributed to writing the manuscript. DRO'D was part of the clinical care team, and contributed to writing the manuscript. WK was part of the clinical care team and contributed to writing the manuscript. SJP conceived and supervised the study, and contributed to writing the manuscript. MET conceived and supervised the study, led the infectious diseases consultation service, collected the data, contributed to data analysis, and writing the manuscript. All authors approved the final manuscript.

References

1. Burke RE, Halpern MS, Baron EJ, Gutierrez K. Pediatric and neonatal *Staphylococcus aureus* bacteremia: epidemiology, risk factors, and outcome. Infection Control and Hospital Epidemiology 2009;30(7):636–44.

2. Mylotte JM, Tayara A. *Staphylococcus aureus* bacteremia: predictors of 30-day mortality in a large cohort. Clinical Infectious Diseases 2000;31(5):1170–4.

3. Healy CM, Palazzi DL, Edwards MS, Campbell JR, Baker CJ. Features of invasive staphylococcal disease in neonates. Pediatrics 2004;114(4):953–61.

4. Frederiksen MS, Espersen F, Frimodt-Møller N, et al. Changing epidemiology of pediatric *Staphylococcus aureus* bacteremia in Denmark from 1971 through 2000. The Pediatric Infectious Disease Journal 2007;26(5):398–405.

5. Hakim H, Mylotte JM, Faden H. Morbidity and mortality of Staphylococcal bacteremia in children. American Journal of Infection Control 2007;35(2):102–5.

6. Denniston S, Riordan FAI. *Staphylococcus aureus* bacteraemia in children and neonates: a 10 year retrospective review. The Journal of Infection 2006;53(6):387–93.

7. Hill PC, Wong CGS, Voss LM, et al. Prospective study of 125 cases of *Staphylococcus aureus* bacteraemia in children in New Zealand. The Pediatric Infectious Disease Journal 2001;20(9):868–73.

For peer review only

8. Gray J, O'Donoghue B. Bacteraemia with meticillin-susceptible *Staphylococcus aureus* in an English children's hospital. The Journal of Hospital Infection 2011;78(2):158–9.

9. Groome MJ, Albrich WC, Wadula J, Khoosal M, Madhi SA. Community-onset *Staphylococcus aureus* bacteraemia in hospitalised African children: high incidence in HIV-infected children and high prevalence of multidrug resistance. Paediatrics and International Child Health 2012;32(3):140–6.

10. Suryati BA, Watson M. *Staphylococcus aureus* bacteraemia in children: a 5-year retrospective review. Journal of Paediatrics and Child Health 2002;38(3):290–4.

11. Wyllie DH, Crook DW, Peto TEA. Mortality after *Staphylococcus aureus* bacteraemia in two hospitals in Oxfordshire, 1997-2003: cohort study. BMJ Online First 2006;333(7562):281.

12. Friendland IR, du Plessis J, Cilliers A. Cardiac complications in children with *Staphylococcus aureus* bacteremia. The Journal of Pediatrics 1995;127(5):476–8.

13. Carrillo-Marquez M a, Hulten KG, Mason EO, Kaplan SL. Clinical and molecular epidemiology of *Staphylococcus aureus* catheter-related bacteremia in children. The Pediatric Infectious Disease Journal 2010;29(5):410–4.

14. Fowler VG, Sanders LL, Sexton DJ, et al. Outcome of *Staphylococcus aureus* bacteremia according to compliance with recommendations of infectious diseases specialists: experience with 244 patients. Clinical Infectious Diseases 1998;27(3):478–86.

15. Lillie P, Moss P, Thaker H, et al. Development, impact and outcomes of the Hull Bacteraemia Service. QJM 2008;101(11):889–98.

16. Nagao M, Iinuma Y, Saito T, et al. Close cooperation between infectious disease physicians and attending physicians can result in better management and outcome for patients with *Staphylococcus aureus* bacteraemia. Clinical Microbiology and Infection 2010;16(12):1783–8.

17. Robinson JO, Pozzi-Langhi S, Phillips M, et al. Formal infectious diseases consultation is associated with decreased mortality in *Staphylococcus aureus* bacteraemia. European Journal of Clinical Microbiology & Infectious Diseases 2012;31(9):2421–8.

18. Jenkins TC, Price CS, Sabel AL, Mehler PS, Burman WJ. Impact of routine infectious diseases service consultation on the evaluation, management, and outcomes of *Staphylococcus aureus* bacteremia. Clinical Infectious Diseases 2008;46(7):1000–8.

19. Choi S-H, Cho SY, Park J-H, Chung J-W. Impact of infectious-disease specialist consultations on outcomes of *Staphylococcus aureus* bacteremia in a

For peer review only

hospital with a low volume of patients with S. aureus bacteremia. The Journal of Infection 2011;62(2):181–5.

20. Kaech C, Elzi L, Sendi P, et al. Course and outcome of *Staphylococcus aureus* bacteraemia: a retrospective analysis of 308 episodes in a Swiss tertiary-care centre. Clinical Microbiology and Infection 2006;12(4):345–52.

21. Honda H, Krauss MJ, Jones JC, Olsen M a, Warren DK. The value of infectious diseases consultation in *Staphylococcus aureus* bacteremia. The American Journal of Medicine 2010;123(7):631–7.

22. Rieg S, Peyerl-Hoffmann G, de With K, et al. Mortality of S. aureus bacteremia and infectious diseases specialist consultation--a study of 521 patients in Germany. The Journal of Infection 2009;59(4):232–9.

23. Jenkins TC, Price CS, Sabel AL, Mehler PS, Burman WJ. Impact of routine infectious diseases service consultation on the evaluation, management, and outcomes of *Staphylococcus aureus* bacteremia. Clinical Infectious Diseases 2008;46:1000–8.

24. De La Blanchardière A, Boutemy J, Thibon P, Michon J, Verdon R, Cattoir V. Clinical benefit of infectious diseases consultation: a monocentric prospective cohort study. Infection 2012;40(5):501–7.

25. Forsblom E, Ruotsalainen E, Ollgren J, Järvinen A. Telephone Consultation Cannot Replace Bedside Infectious Disease Consultation in the Management of *Staphylococcus aureus* Bacteremia. Clinical Infectious Diseases 2013;56(4):527–35.

26. Tissot F, Calandra T, Prod'hom G, et al. Impact of infectious diseases consultation on outcome of methicillin-resistant *Staphylococcus aureus* bacteraemia in a tertiar centre: a ten-year experience. 21st ECCMID/27th ICC 2010;:S420–421.

27. Minton J, Clayton J, Sandoe J, Mc Gann H, Wilcox M. Improving early management of bloodstream infection: a quality improvement project. BMJ 2008;336(7641):440–3.

28. Lundberg J, Nettleman MD, Costigan M, Bentler S, Dawson J, Wenzel RP. *Staphylococcus aureus* bacteraemia: The cost effectiveness of long-term therapy associated with infectious diseases consultation. Clinical Performance and Quality in Health Care 1998;6:9–11.

29. Enoch DA, Cooke FJ, Guha S, Brown NM. Thermostable nuclease: a study of clinical impact. The Journal of Antimicrobial Chemotherapy 2008;61(3):754–5.

30. BSAC. BSAC Methods for Antimicrobial Susceptibility Testing, Version 12 May 2013. http://bsac.org.uk/wp-content/uploads/2012/02/Version-12-Apr-2013_final.pdf.

For peer review only

31. Libman H, Arbeit R. Complications associated with *Staphylococcus aureus* bacteraemia. Archives of Internal Medicine 1984;144(3):541–5.

32. Appendix S, January F. CDC / NHSN Surveillance Definition of Healthcare-Associated Infection and Criteria for Specific Types of Infections in the Acute Care Setting. 2012;(January).

33. Li JS, Sexton DJ, Mick N, et al. Proposed modifications to the Duke criteria for the diagnosis of infective endocarditis. Clinical Infectious Diseases 2000;30(4):633–8.

34. Durack DT, Lukes AS, Bright DK. New Criteria for Diagnosis of Infective Endocarditis□: Utilization of Specific Echocardiographic Findings. The American Journal of Medicine 1994;96:200–9.

35. Friedman ND, Kaye KS, Stout JE, et al. Health Care–Associated Bloodstream Infections in Adults: A Reason To Change the Accepted Definition of Community-Acquired Infections. Annals of Internal Medicine 2002;10(137):791–7.

36. Liu C, Bayer A, Cosgrove SE, et al. Clinical practice guidelines by the Infectious Diseases Society of America for the treatment of methicillin-resistant *Staphylococcus aureus* infections in adults and children. Clinical Infectious Diseases 2011;52(3):e18–55.

37. Fowler VG, Olsen MK, Corey GR, et al. Clinical identifiers of complicated *Staphylococcus aureus* bacteraemia. Archives of Internal Medicine 2003;163:2066–72.

38. Mermel LA, Allon M, Bouza E, et al. Clinical practice guidelines for the diagnosis and management of intravascular catheter-related infection: 2009 Update by the Infectious Diseases Society of America. Clinical Infectious Diseases 2009;49(1):1–45.

39. Habib G, Hoen B, Tornos P, et al. Guidelines on the prevention, diagnosis, and treatment of infective endocarditis (new version 2009): the Task Force on the Prevention, Diagnosis, and Treatment of Infective Endocarditis of the European Society of Cardiology (ESC). Endorsed by the European. European Heart Journal 2009;30(19):2369–413.

40. Calain P, Krause KH, Vaudaux P, et al. Early termination of a prospective, randomized trial comparing teicoplanin and flucloxacillin for treating severe staphylococcal infections. The Journal of Infectious Diseases 1987;155(2):187–91.

41. Valente AM, Jain R, Scheurer M, et al. Frequency of infective endocarditis among infants and children with *Staphylococcus aureus* bacteremia. Pediatrics 2005;115(1):e15–9.

For peer review only

42. Ross AC, Toltzis P, O'Riordan MA, et al. Frequency and risk factors for deep focus of infection in children with *Staphylococcus aureus* bacteremia. The Pediatric Infectious Disease Journal 2008;27(5):396–9.

43. Johnson AP, Sharland M, Goodall CM, et al. Enhanced surveillance of methicillin-resistant *Staphylococcus aureus* (MRSA) bacteraemia in children in the UK and Ireland. Archives of Disease in Childhood 2010;95(10):781–5.

44. Le Saux N, Howard A, Barrowman NJ, Gaboury I, Sampson M, Moher D. Shorter courses of parenteral antibiotic therapy do not appear to influence response rates for children with acute hematogenous osteomyelitis: a systematic review. BMC infectious diseases 2002;2(16).

For peer review only

Table 1: Baseline characteristics of children with *Staphylococcus aureus* bacteraemia included in the study

Baseline variable	Pre-IDC group N=28 patients (%)	IDC group N=35 patients (%)	Combined N=63 patients (%)
Male	20 (71.4)	20 (57.1)	40 (63.5)
Female	8 (28.6)	15 (42.9)	23 (36.5)
Median age in years (IQR)	4.3 (0.2 – 9.4)	3.4 (0.2 – 12.2)	3.4 (0.2 – 10.7)
Neonates	6 (20)	9 (25.0)	15 (23.8)
Prematurity	4 (66.7)	9 (100.0)	13 (86.7)
Median age in days (IQR)	11 (7 – 47)	26 (23 – 38)	25 (14 – 47)
Median birth weight in grams (IQR)	1117 (630-3535)	820 (755-1120)	830 (710 – 1330)
Congenital heart disease	5 (17.9)	8 (22.9)	13 (20.6)
Chronic Pulmonary Disease	3 (10.7)	5 (14.3)	8 (12.7)
Liver Disease	0	1 (2.9)	1 (1.6)
Malignancy	7 (25.0)	9 (25.7)	16 (25.4)
Metastatic cancer	2 (7.1)	1 (2.9)	3 (4.8)
Neurological condition	4 (14.3)	13 (37.1)	17 (27.0)
Diabetes mellitus	0	1 (2.9)	1 (1.6)
Skin Condition	3 (10.7)	1 (2.9)	4 (6.4)
Atopic dermatitis	3 (100.0)		3 (75.0)
Immunosuppression	6 (21.4)	8 (22.9)	14 (22.2)
Corticosteroid therapy	2	3	5
Anti-neoplastic	5	5	10
Neutropenia	2	0	2
	**N=30 episodes (%)**	**N=36 episodes (%)**	**N=66 episodes (%)**
Mode of Acquisition			
Community-acquired	11 (36.7)	10 (27.8)	21 (31.8)
Healthcare-associated	9 (30.0)	7 (19.4)	16 (24.2)
Hospital-acquired	10 (33.3)	19 (52.8)	29 (43.9)
Duration of symptoms of bacteremia (hours)			
0-24	21 (70.0)	18 (50.0)	39 (59.1)
25-72	1 (3.3)	6 (16.7)	7 (10.6)
>72	7 (23.3)	10 (27.8)	17 (25.8)
Unknown	1 (3.3)	2 (5.6)	3 (4.6)
Organism			
MSSA	28 (93.3)	33 (91.7)	61 (92.4)
MRSA	2 (6.7)	3 (8.3)	5 (7.6)
C-Reactive Protein (nmol/L)	333 (114 – 890)	448 (181 – 1081)	390 (133 –1005)
White cell count (10^9/L)	8.8 (5.8 – 15.6)	10.1 (6.9 – 18.4)	9.6 (6.3 – 16.5)
Neutrophils (x 10^9/L)	5.4 (3.6 – 9)	6.3 (4.9-11.8)	5.7 (3.6 – 11.8)
Platelets (x 10^9/L)	277 (151 – 374)	213 (101 – 270)	224 (140 – 301)

IDC = infectious diseases consultation; IQR = interquartile range; MSSA = methicillin-susceptible *Staphylococcus aureus*; MRSA = methicillin-resistant *Staphylococcus aureus*

For peer review only

Table 2: Clinical features of *Staphylococcus aureus* bacteraemia in children included in the study

Focus of infection at time of bacteremia	Pre-IDC group N=30 episodes (%)	IDC group N=36 episodes (%)	Combined N=66 episodes (%)	P-value
Unknown focus	7 (23.3)	2 (5.6)	9 (13.6)	NS
Intravascular catheter	8 (26.7)	22 (61.3)	30 (45.5)	0.013
Culture confirmed	5 (62.5)	11 (50.0)	16 (53.3)	NS
Thrombophlebitis	0	2 (5.7)	2 (3.0)	NS
Bone/Joint infection	8 (26.7)	8 (22.2)	16 (24.2)	NS
Culture confirmed	4 (50.0)	2 (25.0)	6 (37.5)	NS
Lung	1 (3.3)	1 (2.8)	2 (3.0)	NS
Culture confirmed	0	1 (100.0)	1 (50)	NS
Skin & Soft tissue	7 (23.3)	9 (25.0)	16 (24.2)	NS
Culture confirmed	4 (57.1)	8 (88.9)	12 (75.0)	NS
Deep tissue abscess	2 (6.7)	1 (2.8)	3 (4.6)	NS
Culture confirmed	2 (100.0)	0	2 (66.7)	NS
Other focus	2 (6.7)	4 (11.1)	6 (9.1)	NS
Defervescence at 72 hours				
Yes	18 (60.0)	20 (55.6)	38 (57.6)	NS
No	11 (36.7)	14 (38.9)	25 (37.9)	
Unknown	1 (3.3)	2 (5.6)	3 (4.6)	

IDC = infectious diseases consultation; NS = non-significant

For peer review only

Table 3: Risk factors for *Staphylococcus aureus* bacteraemia in children included in the study

Risk factor for SAB	Pre-IDC group N=30 episodes (%)	IDC group N=36 episodes (%)	All children N=66 (episodes)	P-value
Age <1 year	11 (36.7)	14 (38.9)	25 (37.9)	NS
Underlying medical condition	16 (53.3)	24 (66.7)	40 (60.6)	NS
Duration in hospital in days (IQR)<br>Prior to bacteremia*	11.5 (7.0-21.0)	19.0 (12.0-37.0)	16.0 (8.0-24.0)	NS
Prosthetic material	14 (46.7)	26 (72.2)	40 (60.6)	0.04
Intravascular line	13 (43.3)	23 (63.9)	36 (54.6)	NS
Endotracheal tube	0	4	4	NS
Other	1	6	8	NS
Corticosteroid therapy	2 (6.7)	3 (8.3)	5 (7.6)	NS
Surgery within previous 30 days	4 (13.3)	4 (11.1)	8 (12.1)	NS

IDC=infectious disease consultation; IQR=interquartile range; NS = non-significant; SAB=*Staphylococcus aureus* bacteremia
*Hospital-acquired infection only

For peer review only

Table 4: Comparison of the management and outcome of *Staphylococcus aureus* bacteraemia in children, pre- and post-introduction of an infectious disease consult service.

Quality indicator	Pre-IDC group N=30 episodes (%)	IDC group N=36 episodes (%)	P-Value
Median time to infectious diseases review in days (IQR)	N=4<br>3.5 (0.5-21.5)	N=34<br>2.0 (1.0-4.0)	NS
Repeat blood culture performed	26 (86.7)	32 (88.9)	NS
Time to repeat blood culture			
0-48 hours	20	18	NS
48-96 hours	8	13	NS
> 96 hours	1	1	NS
Repeat blood culture positive	6	6	NS
0-48 hours	3	4	NS
48-96 hours	0	0	NS
> 96 hours			
Echocardiogram performed			
Yes	10 (33.3)	29 (80.6)	0.0001
No	20 (66.7)	7 (19.4)	
Beta-Lactam therapy	27 (90.0)	34 (94.4)	NS
MSSA	25 (92.6)	33 (97.1)	
Removable focus of infection	7 (23.3)	22 (43.9)	0.003
Focus removed	6 (85.7)	21 (95.5)	NS
Median time to removal in days (IQR)	2.0 (2.0-2.0)	3.0 (1.0-18.0)	NS
Median time to appropriate antibiotics in days (IQR)	0.0 (0)	0.0 (0)	NS
Median duration of IV antibiotics in days (IQR)	13.5 (7.0-21.0)	18.0 (15.0-29.0)	0.035
Median duration of IV and/or oral antibiotics in days (IQR)	20.5 (16.0-42)	19.0 (15.0 – 29.5)	NS
**Complicated infection**	**N=15 episodes Days (IQR)**	**N=19 episodes Days (IQR)**	
Median duration of IV antibiotics	14.0 (6.0-21.0)	22 (15.0-39.0)	0.02
Median duration of IV or oral antibiotics	19.0 (17.0-43.0)	27.0 (16.0-39.0)	NS
Median duration of IV antibiotics if repeat blood culture positive	13.0 (6.0 – 14.0)	19.0 (15.0 – 27.0)	0.007
Met standard recommendation 28 days IV antibiotics (%)	2 (13.3)	8 (42.1)	NS
**Uncomplicated infection**	**N=15 episodes Days (IQR)**	**N=17 episodes Days (IQR)**	
Median duration of IV antibiotics	13.0 (7.0 – 22.0)	15.0 (14.0-21.0)	NS
Median duration of IV or oral antibiotics (IQR)	22.0 (14.0-32.0)	18.0 (14.0-29.0)	NS
Met standard			

For peer review only

recommendation of 14 days (%)	7 (46.7)	13 (68.4)	NS
**Outcomes**	**N=30 episodes Days (IQR)**	**N=36 episodes Days (IQR)**	
Median duration of hospital admission<br>Total<br>Complicated<br>Uncomplicated	 <br>14.0 (6.0-42.0)<br>20.0 (6.0 – 49.0)<br>7.0 (3.0-22.0)	 <br>16.5 (7.5-58)<br>25.0 (8.0-88.0)<br>11.0 (6.0-36.0)	 <br>NS<br>NS<br>NS
SAB recorded in discharge summary	24 (80.0)	29 (82.9)	NS
Secondary infection detected	1	3	NS
Outcomes 30-day post-SAB<br>Death<br>Recurrence	 0<br>0	 3<br>0	 NS<br>NS
Outcomes 30-90 days post SAB<br>Death<br>Recurrence	 2<br>2	 0<br>1	 NS<br>NS

IDC = infectious diseases consultation; IQR = Interquartile range; IV = intravenous; NS = non-significant; SAB = *Staphylococcus aureus* bacteremia

For peer review only

Figure 1. Study schema of paediatric patients with *S. aureus* bacteraemia

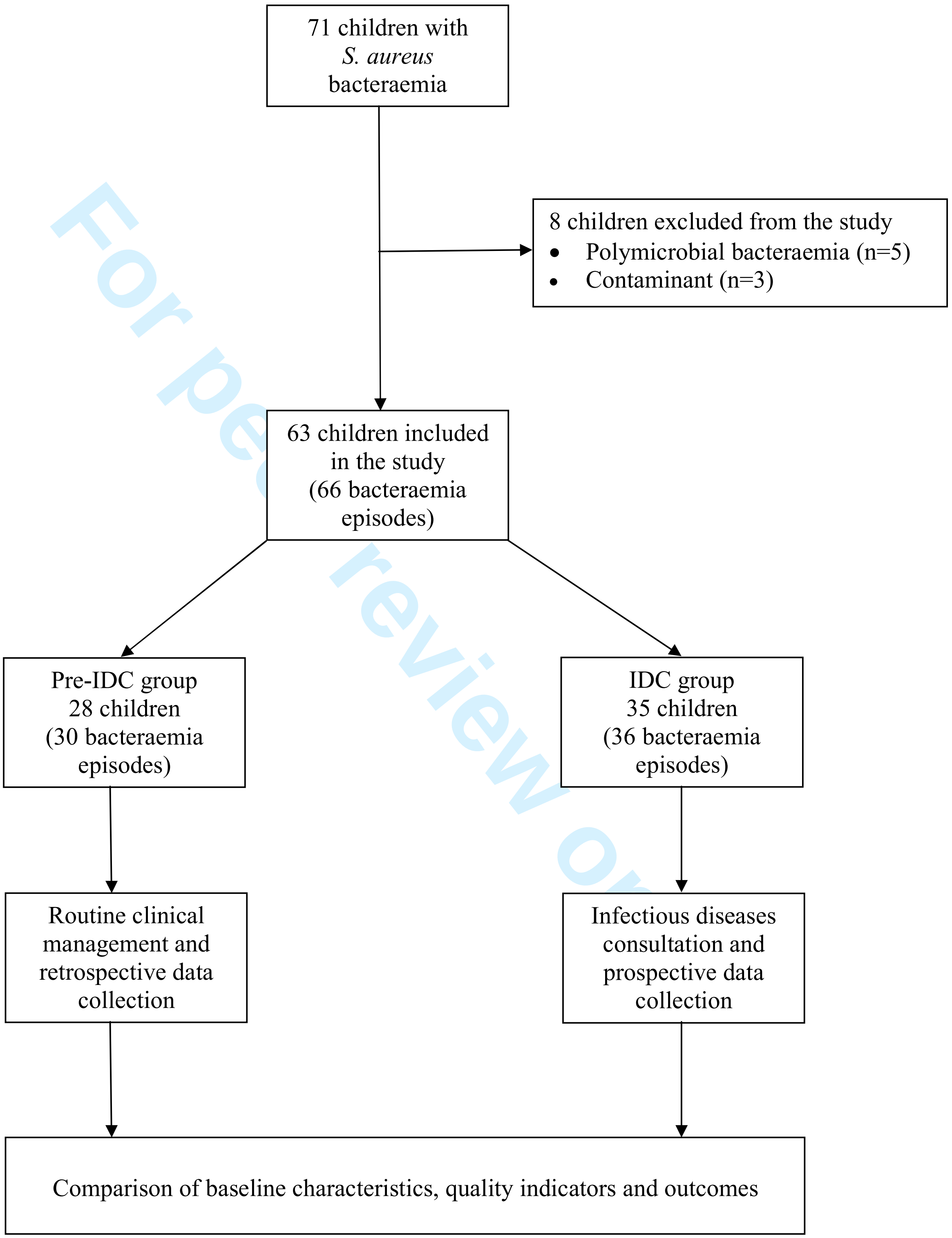

For peer review only - http://bmjopen.bmj.com/site/about/guidelines.xhtml

BMJ Open

Impact of infectious diseases consultation on the management of S. aureus bactaeremia in children

Journal:	*BMJ Open*
Manuscript ID:	bmjopen-2013-004659.R1
Article Type:	Research
Date Submitted by the Author:	02-Jun-2014
Complete List of Authors:	Saunderson, Rebecca; University of Cambridge, Department of Medicine<br>Gouliouris, Theodore; University of Cambridge, Department of Medicine<br>Cartwright, Edward; University of Cambridge, Department of Medicine<br>Nickerson, Emma; Cambridge University Hospitals NHS Foundation Trust, Department of Infectious Diseases<br>Aliyu, Sani; Cambridge University Hospitals NHS Foundation Trust, Department of Infectious Diseases<br>O'Donnell, Roddy; Cambridge University Hospitals NHS Foundation Trust, Department of Paediatrics<br>Kelsall, Wilf; Cambridge University Hospitals NHS Foundation Trust, Department of Paediatrics<br>Limmathurotsakul, Direk; Mahidol Oxford Research Unit,<br>Peacock, Sharon; University of Cambridge, Department of Medicine<br>Torok, Estee; University of Cambridge, Department of Medicine
**Primary Subject Heading**:	Infectious diseases
Secondary Subject Heading:	Epidemiology, Medical management
Keywords:	Epidemiology < INFECTIOUS DISEASES, Diagnostic microbiology < INFECTIOUS DISEASES, Cardiology < INTERNAL MEDICINE

SCHOLARONE™
Manuscripts

Title

Impact of infectious diseases consultation on the management of *S. aureus* bacteraemia in children

Authors

Rebecca B. Saunderson,[1] Theodore Gouliouris,[1,2,3] Edward J. Cartwright,[1,3] Emma J. Nickerson,[4] Sani H. Aliyu,[2,4] D. Roddy O'Donnell,[5] Wilf Kelsall,[5] D. Limmathurotsakul,[7] Sharon J. Peacock,[1,2,3,6] M. Estée Török[1,2,3]

Affiliations

1. Department of Medicine, University of Cambridge, Cambridge, United Kingdom

2. Department of Microbiology, Cambridge University Hospitals NHS Foundation Trust, Cambridge, United Kingdom

3. Public Health England, Clinical Microbiology and Public Health Laboratory, Cambridge, United Kingdom

4. Department of Infectious Diseases, Cambridge University Hospitals NHS Foundation Trust, Cambridge, United Kingdom

5. Department of Paediatrics, Cambridge University Hospitals NHS Foundation Trust, Cambridge, United Kingdom

6. Wellcome Trust Sanger Institute, Hinxton, United Kingdom

7. Mahidol Oxford Research Unit, Mahidol University, Bangkok, Thailand

Correspondence:

Dr M. Estée Török

For peer review only

University of Cambridge, Department of Medicine, Box 157, Addenbrooke's Hospital, Hills Road, Cambridge CB2 0QQ, United Kingdom

Telephone: +44 (0) 1223 336 845

Facsimile: +44 (0) 1223 336 846

Email: et317@medschl.cam.ac.uk

Key words

Staphylococcus aureus bacteraemia; infectious diseases consultation; management; outcome; paediatric

Word count

Abstract = 260 words; Main text = 3,605; Tables = 4; Figures=1

For peer review only

ABSTRACT

Objectives

Infectious diseases consultation (IDC) in adults with *Staphylococcus aureus* bacteraemia (SAB) has been shown to improve management and outcome. The aim of this study was to evaluate the impact of IDC on the management of SAB in children.

Study design

Observational cohort study of children with SAB.

Setting

Cambridge University Hospitals NHS Foundation Trust, a large acute NHS Trust in the United Kingdom.

Participants

All children with SAB admitted to the Cambridge University Hospitals NHS Foundation Trust between 16 July 2006 and 31 December 2012.

Methods

Children with SAB between 2006 and 31 October 2009 were managed by routine clinical care (pre-IDC group) and data were collected retrospectively by case notes review. An IDC service for SAB was introduced in November 2009. All children with SAB were reviewed regularly and data were collected prospectively (IDC group) until 31 December 2012. Baseline characteristics, quality metrics, and outcome were compared between the pre-IDC group and IDC group.

Results

There were 66 episodes of SAB in 63 children – 28 patients (30 episodes) in the pre-IDC group, and 35 patients (36 episodes) in the IDC group. The median age was 3.4 years (IQR 0.2 – 10.7 years). Patients in the IDC group were more likely to have echocardiography performed, a removable focus of infection identified, and to receive

For peer review only

a longer course of intravenous antimicrobial therapy. There were no differences in total duration of antibiotic therapy, duration of hospital admission, or outcome at 30 or 90 days following onset of SAB.

Conclusions

IDC resulted in improvements in the investigation and management of SAB in children.

For peer review only

ARTICLE SUMMARY

Strengths and limitations of the study

- This is the first study to examine the impact of introduction of infectious diseases consultation (IDC) on the management of *S. aureus* bacteraemia (SAB) in children.
- We found that IDC was associated with an improvement in investigation and management of SAB, but there was no difference in mortality between the pre-IDC and IDC groups.
- The main limitation of the study was the size of the study population, which may explain the lack of mortality benefit.
- The study was conducted in a tertiary referral centre, where clinical management is likely to have been good prior to introduction of the IDC, and may not be generalizable to other settings.

For peer review only

MAIN TEXT

Introduction

Staphylococcus aureus bacteraemia (SAB) is a serious infection that leads to significant morbidity and mortality in adults and children.[1,2] *Staphylococcus aureus* (*S. aureus*) causes significant disease in the paediatric population, occurring in 1.5% of all neonatal intensive care unit (ICU) admissions[3], and 6 per 100,000 children older than 1 year of age.[4] In neonates, SAB is almost always hospital-acquired, and is frequently due to intravascular catheter (IVC) associated infections.[3–6] The majority of non-neonatal cases of SAB are community-acquired; those that are hospital-acquired infections are usually IVC-associated.[7,8]

Identified risk factors for the development of SAB in the paediatric population include having a pre-existing medical condition, prolonged hospitalization, the presence of an IVC, and HIV infection.[1,3–6,9,10] Mortality from SAB in the adult population is about 30% .%.[11] Mortality rates in the paediatric population tend to be lower, but can be up to 15% in neonates and/or children with co-morbidities.[1,5,9,12,13] Given that SAB causes a substantial burden of disease in the paediatric population, strategies to improve management, prevent the complications of SAB, and reduce mortality are a clinical priority.

The impact of infectious disease consultation (IDC) in adults with SAB has been extensively studied . .[14–24] IDC has been associated with improved adherence to guidelines, including appropriate and targeted investigation, optimal duration of antibiotic therapy, and a reduction in complicated infection, morbidity and mortality.[14,16,17,22,23,25–28] In contrast, the impact of an IDC on the management and

For peer review only

outcomes of SAB in children has not previously been evaluated. The aim of this study was to determine the effects of routine IDC on the investigation, management, and outcome of children with SAB.

Materials and methods

Study setting and participants

Cambridge University Hospitals National Health Service Foundation Trust (CUH) is a tertiary referral centre for paediatrics in the East of England. The paediatric service has a 22-bed medical and surgical ward, a 17-bed paediatric haematology and oncology ward, an 11-bed paediatric ICU and high dependency unit (both caring for children aged from 0 to 16 years), and a 12-bed surgical and medical ward for children aged up to 3 years. The Rosie Hospital, the on-site mother and baby hospital, has a 17-cot Neonatal ICU and a 10-cot Special Care Baby Unit.

Study design

We conducted an observational cohort study of all children with SAB admitted to CUH between 16 July 2006 and 31 December 2012. In November 2009 an IDC service for all patients with SAB was established at CUH. Data were collected from 2006 to 2009 by a retrospective review of the medical records, and prospectively thereafter during the IDC service. Patients with blood cultures that were considered to be contaminants (afebrile with no clinical evidence of infection) or with polymicrobial blood cultures were excluded from the analysis.

Microbiological investigation

Blood cultures were collected and incubated at 37°C for 5 days using BacT/Alert 3D

For peer review only

system (bioMérieux, Basingstoke, UK). Blood cultures that flagged positive were examined by microscopy and presumptively identified as *S. aureus* using a thermostable nuclease test.[29] Colonies of *S. aureus* were identified by routine methods after a further overnight incubation. Identification of *S. aureus* was performed using a commercial latex agglutination test (Staphaurex, Remel, Lenexa, USA) until 2011 and then using matrix-assisted laser desorption ionization time-of-flight mass spectroscopy (Bruker Daltonik, Bremen, Germany). Antibiotic susceptibilities were determined using disc diffusion testing, according to British Society for Antimicrobial Chemotherapy standards.[30] Throughout the study period a clinical microbiologist provided telephone advice to the clinical team for all patients with SAB, and attended weekly ward rounds on the paediatric oncology ward and paediatric ICU.

Study procedures

Prior to November 2009 all patients with *S. aureus* bacteraemia were managed by their primary clinical care team, with telephone advice from the microbiologists. From November 2009, all patients with SAB were reviewed by an infectious diseases Specialist Registrar or Consultant, following presumptive identification of *S. aureus* in blood cultures. The assessment included clinical history to determine symptoms of infection, and physical examination to determine possible foci of infection. Patients underwent clinical review daily by their primary care team and at least weekly by the IDC team during their inpatient stay. Demographic, clinical and microbiology data were collected using a standard case record form, and entered into an electronic database.

An IVC was considered to be the focus of infection if there was evidence of

For peer review only

inflammation at the catheter exit site and/or a vascular catheter tip culture positive for *S. aureus,* without clinical evidence of another source of bacteraemia.[31] Thrombophlebitis was diagnosed when there was clinical evidence of infection and inflammation along a blood vessel or when ultrasound or other imaging confirmed the presence of intravascular thrombosis in the setting of suspected infection. Bone and joint infections were defined according to United States Centers for Disease Control and Prevention criteria.[32] The lung was considered to be the source of infection when there was clinical, radiological and/or microbiological evidence of pulmonary infection. Soft tissue infection was considered to be the source of the bacteraemia if the clinical signs of a known or suspected soft tissue infection pre-dated or were present at the time of bacteremia.[14] A deep tissue abscess was defined by radiological imaging criteria. Infective endocarditis (IE) was diagnosed according to the modified Duke criteria.[33,34]

A SAB episode was defined as being greater than or equal to 14 days from a previous episode, in the absence of persistent bacteraemia or focus of infection. A secondary site of infection was defined as a site of infection separate from the primary site of infection that was not present at the time of the initial examination. Healthcare-associated bloodstream infection was defined according to previously published criteria.[35] Hospital-acquired infection was defined according to United States Centers for Disease Control and Prevention ..[32] Community-acquired infections were defined as those patients with a positive blood culture taken at or within 48-hours of admission who did not meet criteria for healthcare-associated bloodstream infection.[35] Patients were classified as having uncomplicated SAB if blood cultures were negative two to four days after the initial blood culture was positive, if they had defervesced at

For peer review only

72 hours, if there was no evidence of metastatic disease or endocarditis, or if they had a catheter related infection.[36]

Appropriate antimicrobial therapy was defined as therapy to which the isolate was determined to be susceptible by antimicrobial disc susceptibility testing. The duration of therapy was the length of time that a patient received antibiotics to which the isolate was susceptible. An underlying medical condition was defined as any chronic medical condition that was present at the time of bacteraemia. Serum C-reactive protein (CRP), blood white cell counts, and platelet counts were measured on the day of, or within 48 hours post -bacteraemia. Duration of hospital admission and outcome at 30 and 90 days post-bacteraemia were recorded for all patients.

Treatment recommendations

Antimicrobial treatment recommendations were provided for all children with SAB, based on existing evidence on the management of SAB in adults.[14,37–40] These included removal of a removable focus of infection,[14] performing repeat blood cultures at 48 to 96 hours,[37] performing a transthoracic echocardiogram, performing radiological imaging of suspected deep foci of infection, treating uncomplicated infection with 14 days of intravenous (IV) antibiotics,[38] treating complicated infections with a minimum of 28 days of IV antibiotics[39], and using beta-lactam therapy as the mainstay of treatment for methicillin-susceptible *S. aureus*. .[40]

Statistical analysis

Data were analyzed using STATA version 12 (StataCorp, College Station, Texas, USA). Categorical variables were analyzed using the Fisher's exact test and reported

For peer review only - http://bmjopen.bmj.com/site/about/guidelines.xhtml

as the number and percent. Continuous variables were compared using the Mann Whitney U test and reported as the median and interquartile range. Mortality was analysed per patient (i.e. only the first bacteraemia episode was analysed).

Ethics statement

Written informed consent from participants was not required as the study was conducted as a service evaluation. The study protocol received approval from by the University of Cambridge Human Biology Research Ethics Committee, and the CUH Research and Development Department.

Results

Patient characteristics

Between July 2006 to December 2012, 71 children had one or more blood cultures that were positive for *S. aureus.* Sixty-three children (66 episodes) were included in the study. Five children (six episodes) were excluded because of polymicrobial bacteraemia and Three patients (three episodes) were excluded because the cultures were considered to be contaminants. Thus, 28 patients (30 episodes) were included in the pre-IDC group, and 35 patients (36 episodes) in the IDC group. The study schema is summarized in Figure 1. Four of 30 episodes (13.3%) received an IDC before the service was implemented in 2009, and 34 of 36 episodes (94.4%) received an IDC after the service was implemented in 2009.

The baseline characteristics of the two groups were similar (Table 1). The clinical features for SAB were likewise similar, apart from an increased proportion of IVC-related infections in the IDC group (61.3% versus 26.7%, P<0.01) (Table 2). A higher

For peer review only

proportion of patients had an unidentified focus of infection in the pre-IDC group compared with the IDC group (23.3% versus 5.6%, P=0.07). risk factors for SAB were also similar apart from an increased frequency of prosthetic material in the IDC group (72.2% versus 46.7%, P=0.04) (Table 3).

Clinical management

A service evaluation of the IDC service was conducted, the results of which are summarized in Table 4. In the IDC group, 34/36 episodes had an infectious diseases review, with a median time to review of two days (range 1 to 4 days). Patients in the IDC group were more likely to have transthoracic echocardiography performed (80.6% versus 33.3%, P<0.01). They were also more likely to have a removable focus of infection identified (43.9% versus 23.3%, P<0.01), although there was no difference between the two groups in the likelihood of removal, or the time to removal. In the IDC group two patients did not have their IVC removed, despite the recommendation to do so, because of concerns about difficulty in re-establishing vascular access. There was no difference in the number of repeat blood cultures performed between groups.

Antimicrobial therapy

There was no difference between the two groups in the time taken to initiate appropriate antimicrobial therapy. Patients in the IDC group were more likely to receive a longer duration of IV antimicrobial therapy (18 days versus 13.5 days, P=0.04), although there was no difference in total duration of therapy (IV and oral) between the two groups. In patients with complicated SAB, the duration of IV antibiotic therapy was longer in the IDC group (22 days versus 14 days, P=0.02)

For peer review only

although there was no difference in total duration of antibiotic therapy (IV and oral) between the two groups. Patients in the IDC group were more likely to receive a longer duration of IV therapy if their repeat blood culture result was positive (P<0.01). In patients with uncomplicated SAB there was no difference between groups in the duration of IV antibiotics, or the total duration of antibiotic therapy. In terms of compliance with recommended standards for duration of therapy, patients in the IDC group were more likely to meet these standards compared with patients in the non-IDC group, both for complicated SAB (42.1% versus 13.3%, P=0.13) and uncomplicated SAB (68.4% versus 46.7%, P=0.14). There was no difference in the proportion of patients receiving beta-lactam therapy for MSSA bacteraemia between the two groups.

Outcome of SAB

The duration of hospital admission was similar in the pre-IDC and IDC groups, and in those with uncomplicated and complicated SAB (Table 4). SAB was recorded in the discharge summary in the majority of patients in both groups. Four secondary infections were diagnosed, three in the IDC group and one in the pre-IDC group. In the IDC group, the secondary infections were tricuspid valve endocarditis (in a very low birth weight neonate with patent ductus arteriosus), pneumonia, and osteomyelitis, respectively. One child in the non-IDC group developed osteomyelitis. There were three cases of recurrent bacteraemia within 90 days – two in the pre-IDC group and one in the IDC group. Three children died within 30 days of SAB, all in the IDC group, giving an overall 30-day mortality rate of 4.8%. One death occurred in a child with metastatic cancer and was not attributed to the SAB. The other two deaths were deemed attributable to SAB, as blood cultures were positive at the time of death.

For peer review only

One patient was a neonate and died prior to IDC, and the second case had cerebellar atrophy, developmental delay and was receiving total parenteral nutrition. The 90-day mortality rate was 7.9%. Two patients died between 30 and 90 days post-SAB, both in the pre-IDC group. One patient had metastatic cancer, and the other had complex congenital heart disease.

Discussion

To our knowledge this is the first study to systematically examine the impact of introduction of an IDC service on the management of SAB in children. We compared the clinical features, management, and outcomes of all children presenting with SAB to our hospital between July 2006 and December 2012, before and after introduction of an IDC service. The main findings of the study were that patients in the IDC group were more likely to have echocardiography performed, a removable focus of infection identified, and to receive a longer course of IV antibiotic therapy. These findings concur with those from previous studies of IDC conducted in adults with SAB, and reflect current best practice.

Follow-up blood cultures have been recommended in adults, as prolonged bacteraemia is a predictor of complicated infection and poorer outcome in SAB.[37] As a result, prolonged IV antibiotic therapy is recommended in patients with positive repeat blood cultures. We found that children who had a positive repeat blood culture were more likely to receive a longer course of IV antibiotics if they were in the IDC group.

Echocardiography was performed in a higher proportion of children in the IDC group

For peer review only

compared with the pre-IDC group. The rates of IE in children with SAB are reported to be between 0 and 20%, which is similar to rates reported in the adult population.[1,3,6,8,12,41–43] An American study by Valente and colleagues diagnosed IE in 20% of children with SAB (~12% of whom had confirmed IE) .).[41] Children with underlying congenital heart disease had a higher prevalence of confirmed or probable IE compared to those who had structurally normal hearts (53% versus 3%) and patients with definite IE had multiple positive blood cultures. Mortality was higher in patients with endocarditis compared to those without (40% versus 12%). Another study from South Africa reported an IE rate of 11% in children with SAB.[12] Risk factors for the development of IE in children include congenital heart disease, a central IVC, and persistently positive blood cultures after 24 hours. [41],[42] In the United Kingdom, there are no published guidelines on the use of echocardiography in children with SAB. The Infectious Diseases Society of America guidelines for MRSA bacteraemia recommend performing echocardiography in children with congenital heart disease, those with bacteraemia duration greater than two days, or those with other clinical findings suggestive of endocarditis.[36] In our study the one child who developed tricuspid valve endocarditis was a very low birth weight premature neonate with a patent ductus arteriosus, an IVC-related infection, and persistent bacteremia. Our findings concur with these guidelines, and support the use of a risk-based strategy for the use of echocardiography in children with SAB.

We found that a higher proportion of children had an IVC-related infection, and/or a removable focus of infection in the IDC group compared with the pre-IDC group, although neither of these differences were statistically significant. It is possible that IVC was used more during the IDC period compared with the pre-IDC period.

For peer review only

Although removable foci of infection were more frequently identified and removed in the IDC group compared to the pre-IDC group, the median time to removal was slightly longer (3 versus 2 days). In some cases this was related to practical difficulties in removing the focus, such as re-establishing vascular access in neonates. Conversely, there were fewer patients with an unidentified focus of infection in the IDC group compared with the pre-IDC group. These findings indicate that the introduction of specialist IDC service improved the rate of diagnosis of the focus of infection in children with SAB, suggesting that the consult service was beneficial.

The frequency of MRSA bacteraemia was only 7.6% in our study cohort. Possible explanations for this are that MRSA bacteraemia is less common in children than in adults , and that MRSA bacteraemia rates have significantly declined in the United Kingdom since 2001 . By contrast, in other countries such as the United States, the incidence of MRSA bloodstream infections have been higher than in the UK , but have recently declined . In a large retrospective study of over 57,000 hospitalised children with *S. aureus* infections, 51% had MRSA and 61% had MRSA skin and soft tissue infections. The incidence of skin and soft tissue infections, pneumonia, osteomyelitis and bacteraemia increased over time but overall mortality was low (1%) . Thus the findings of our study may not be generalizable to other settings where the epidemiology and outcomes of MRSA bacteraemia are different.

In terms of duration of antimicrobial therapy, we found that patients in the IDC group received longer courses of IV antibiotics in complicated infection, compared with patients in the pre-IDC group. This concurs with findings from studies in adults with SAB, and suggests that specialist infectious diseases review may be beneficial in

For peer review only

ensuring that clinical management recommendations, such as the length of IV antimicrobial therapy, are applied. There were, However, no differences in mortality observed between the pre-IDC and IDC groups. The most likely explanation for this was the small study population, combined with a low mortality rate, which meant that a large number of patients would be required to demonstrate even in small difference in mortality. Furthermore the overall duration of antibiotic therapy was similar in the two groups. It may be that total duration of antibiotic therapy is more important than the route of administration, provided that adequate concentrations are achieved in the bloodstream. Indeed studies to examine this very question, comparing short versus long courses of IV antibiotics in SAB in adults are ongoing. Finally, although a removable focus of infection was identified more frequently in the IDC group, the likelihood of removal and the time to removal did not differ; this may also explain the lack of difference in outcome between the two groups.

We acknowledge several limitations to our study. The study population was small and the differences in diagnosis and management that we observed in the two groups did not translate into differences in outcome, for reasons discussed above. The retrospective data collection during the pre-IDC period (2006 to 2009) carries a risk of incomplete recording of data and potential bias. There were, however, no differences in baseline characteristics between the two groups in terms of age, gender, underlying co-morbidities or focus of infection. The only exception was a higher frequency of IVC-related infections in the IDC group, which may have been under-recorded in the pre-IDC period and / or diagnosed more frequently in the IDC period.

In conclusion, we found that introduction of IDC for paediatric SAB resulted in

For peer review only

improvements in management and a more consistent approach to care across the paediatric service. These findings concur with those of previous studies of IDC in adults with SAB. Our findings support the use of a targeted approach to echocardiography in SAB in children, particularly for patients with risk factors for complicated disease. Despite improvements in the investigation and clinical management, we did not find any differences in the development of secondary infections, recurrent bacteremia, or death between the two groups. The most likely explanation for this is the small study population and larger prospective studies are required validate our findings and to determine the optimal strategies for investigation and management of paediatric SAB.

Funding

This work was supported by grants from the United Kingdom Clinical Research Collaboration (UKCRC) Translational Infection Research Initiative (TIRI); the Medical Research Council (G1000803), with contributions from the Biotechnology and Biological Sciences Research Council, the National Institute for Health Research (NIHR) on behalf of the United Kingdom Department of Health, and the Chief Scientist of the Scottish Government Health Directorate; the Public Health England; and the NIHR Cambridge Biomedical Research Centre. MET is a Clinician Scientist Fellow funded by the Academy of Medical Sciences at the Health Foundation.

Competing interests statement

All authors have no conflicts of interest to declare.

Author contributions

For peer review only

RBS was part of the infectious diseases consultation service, collected the data, performed the data analysis, and wrote the first draft of the manuscript. TG was part of the infectious diseases consultation service, collected the data, and contributed to writing the manuscript. EJC collected the data, and contributed to the data analysis writing the manuscript. EJN was part of the infectious diseases consultation service, collected the data, and contributed to writing the manuscript. SHA was part of the infectious diseases consultation service, collected the data, and contributed to writing the manuscript. DRO'D was part of the clinical care team, and contributed to writing the manuscript. WK was part of the clinical care team and contributed to writing the manuscript. SJP conceived and supervised the study, and contributed to writing the manuscript. MET conceived and supervised the study, led the infectious diseases consultation service, collected the data, contributed to data analysis, and writing the manuscript. All authors approved the final manuscript.

References

1. Burke RE, Halpern MS, Baron EJ, Gutierrez K. Pediatric and neonatal *Staphylococcus aureus* bacteremia: epidemiology, risk factors, and outcome. Infection Control and Hospital Epidemiology 2009;30(7):636–44.

2. Mylotte JM, Tayara A. *Staphylococcus aureus* bacteremia: predictors of 30-day mortality in a large cohort. Clinical Infectious Diseases 2000;31(5):1170–4.

3. Healy CM, Palazzi DL, Edwards MS, Campbell JR, Baker CJ. Features of invasive staphylococcal disease in neonates. Pediatrics 2004;114(4):953–61.

4. Frederiksen MS, Espersen F, Frimodt-Møller N, et al. Changing epidemiology of pediatric *Staphylococcus aureus* bacteremia in Denmark from 1971 through 2000. The Pediatric Infectious Disease Journal 2007;26(5):398–405.

5. Hakim H, Mylotte JM, Faden H. Morbidity and mortality of Staphylococcal bacteremia in children. American Journal of Infection Control 2007;35(2):102–5.

6. Denniston S, Riordan FAI. *Staphylococcus aureus* bacteraemia in children and neonates: a 10 year retrospective review. The Journal of Infection 2006;53(6):387–93.

For peer review only

7. Hill PC, Wong CGS, Voss LM, et al. Prospective study of 125 cases of *Staphylococcus aureus* bacteraemia in children in New Zealand. The Pediatric Infectious Disease Journal 2001;20(9):868–73.

8. Gray J, O'Donoghue B. Bacteraemia with meticillin-susceptible *Staphylococcus aureus* in an English children's hospital. The Journal of Hospital Infection 2011;78(2):158–9.

9. Groome MJ, Albrich WC, Wadula J, Khoosal M, Madhi SA. Community-onset *Staphylococcus aureus* bacteraemia in hospitalised African children: high incidence in HIV-infected children and high prevalence of multidrug resistance. Paediatrics and International Child Health 2012;32(3):140–6.

10. Suryati BA, Watson M. *Staphylococcus aureus* bacteraemia in children: a 5-year retrospective review. Journal of Paediatrics and Child Health 2002;38(3):290–4.

11. Wyllie DH, Crook DW, Peto TEA. Mortality after *Staphylococcus aureus* bacteraemia in two hospitals in Oxfordshire, 1997-2003: cohort study. BMJ Online First 2006;333(7562):281.

12. Friendland IR, du Plessis J, Cilliers A. Cardiac complications in children with *Staphylococcus aureus* bacteremia. The Journal of Pediatrics 1995;127(5):476–8.

13. Carrillo-Marquez M a, Hulten KG, Mason EO, Kaplan SL. Clinical and molecular epidemiology of *Staphylococcus aureus* catheter-related bacteremia in children. The Pediatric Infectious Disease Journal 2010;29(5):410–4.

14. Fowler VG, Sanders LL, Sexton DJ, et al. Outcome of *Staphylococcus aureus* bacteremia according to compliance with recommendations of infectious diseases specialists: experience with 244 patients. Clinical Infectious Diseases 1998;27(3):478–86.

15. Lillie P, Moss P, Thaker H, et al. Development, impact and outcomes of the Hull Bacteraemia Service. QJM 2008;101(11):889–98.

16. Nagao M, Iinuma Y, Saito T, et al. Close cooperation between infectious disease physicians and attending physicians can result in better management and outcome for patients with *Staphylococcus aureus* bacteraemia. Clinical Microbiology and Infection 2010;16(12):1783–8.

17. Robinson JO, Pozzi-Langhi S, Phillips M, et al. Formal infectious diseases consultation is associated with decreased mortality in *Staphylococcus aureus* bacteraemia. European Journal of Clinical Microbiology & Infectious Diseases 2012;31(9):2421–8.

18. Jenkins TC, Price CS, Sabel AL, Mehler PS, Burman WJ. Impact of routine infectious diseases service consultation on the evaluation, management, and

For peer review only

outcomes of *Staphylococcus aureus* bacteremia. Clinical Infectious Diseases 2008;46(7):1000–8.

19. Choi S-H, Cho SY, Park J-H, Chung J-W. Impact of infectious-disease specialist consultations on outcomes of *Staphylococcus aureus* bacteremia in a hospital with a low volume of patients with S. aureus bacteremia. The Journal of Infection 2011;62(2):181–5.

20. Kaech C, Elzi L, Sendi P, et al. Course and outcome of *Staphylococcus aureus* bacteraemia: a retrospective analysis of 308 episodes in a Swiss tertiary-care centre. Clinical Microbiology and Infection 2006;12(4):345–52.

21. Honda H, Krauss MJ, Jones JC, Olsen M a, Warren DK. The value of infectious diseases consultation in *Staphylococcus aureus* bacteremia. The American Journal of Medicine 2010;123(7):631–7.

22. Rieg S, Peyerl-Hoffmann G, de With K, et al. Mortality of S. aureus bacteremia and infectious diseases specialist consultation--a study of 521 patients in Germany. The Journal of Infection 2009;59(4):232–9.

23. Jenkins TC, Price CS, Sabel AL, Mehler PS, Burman WJ. Impact of routine infectious diseases service consultation on the evaluation, management, and outcomes of *Staphylococcus aureus* bacteremia. Clinical Infectious Diseases 2008;46:1000–8.

24. De La Blanchardière A, Boutemy J, Thibon P, Michon J, Verdon R, Cattoir V. Clinical benefit of infectious diseases consultation: a monocentric prospective cohort study. Infection 2012;40(5):501–7.

25. Forsblom E, Ruotsalainen E, Ollgren J, Järvinen A. Telephone Consultation Cannot Replace Bedside Infectious Disease Consultation in the Management of *Staphylococcus aureus* Bacteremia. Clinical Infectious Diseases 2013;56(4):527–35.

26. Tissot F, Calandra T, Prod'hom G, et al. Impact of infectious diseases consultation on outcome of methicillin-resistant *Staphylococcus aureus* bacteraemia in a tertiar centre: a ten-year experience. 21st ECCMID/27th ICC 2010;:S420–421.

27. Minton J, Clayton J, Sandoe J, Mc Gann H, Wilcox M. Improving early management of bloodstream infection: a quality improvement project. BMJ 2008;336(7641):440–3.

28. Lundberg J, Nettleman MD, Costigan M, Bentler S, Dawson J, Wenzel RP. *Staphylococcus aureus* bacteraemia: The cost effectiveness of long-term therapy associated with infectious diseases consultation. Clinical Performance and Quality in Health Care 1998;6:9–11.

For peer review only

29. Enoch DA, Cooke FJ, Guha S, Brown NM. Thermostable nuclease: a study of clinical impact. The Journal of Antimicrobial Chemotherapy 2008;61(3):754–5.

30. BSAC. BSAC Methods for Antimicrobial Susceptibility Testing, Version 12 May 2013. http://bsac.org.uk/wp-content/uploads/2012/02/Version-12-Apr-2013_final.pdf.

31. Libman H, Arbeit R. Complications associated with *Staphylococcus aureus* bacteraemia. Archives of Internal Medicine 1984;144(3):541–5.

32. Appendix S, January F. CDC / NHSN Surveillance Definition of Healthcare-Associated Infection and Criteria for Specific Types of Infections in the Acute Care Setting. 2012;(January).

33. Li JS, Sexton DJ, Mick N, et al. Proposed modifications to the Duke criteria for the diagnosis of infective endocarditis. Clinical Infectious Diseases 2000;30(4):633–8.

34. Durack DT, Lukes AS, Bright DK. New Criteria for Diagnosis of Infective Endocarditis□: Utilization of Specific Echocardiographic Findings. The American Journal of Medicine 1994;96:200–9.

35. Friedman ND, Kaye KS, Stout JE, et al. Health Care–Associated Bloodstream Infections in Adults: A Reason To Change the Accepted Definition of Community-Acquired Infections. Annals of Internal Medicine 2002;10(137):791–7.

36. Liu C, Bayer A, Cosgrove SE, et al. Clinical practice guidelines by the Infectious Diseases Society of America for the treatment of methicillin-resistant *Staphylococcus aureus* infections in adults and children. Clinical Infectious Diseases 2011;52(3):e18–55.

37. Fowler VG, Olsen MK, Corey GR, et al. Clinical identifiers of complicated *Staphylococcus aureus* bacteraemia. Archives of Internal Medicine 2003;163:2066–72.

38. Mermel LA, Allon M, Bouza E, et al. Clinical practice guidelines for the diagnosis and management of intravascular catheter-related infection: 2009 Update by the Infectious Diseases Society of America. Clinical Infectious Diseases 2009;49(1):1–45.

39. Habib G, Hoen B, Tornos P, et al. Guidelines on the prevention, diagnosis, and treatment of infective endocarditis (new version 2009): the Task Force on the Prevention, Diagnosis, and Treatment of Infective Endocarditis of the European Society of Cardiology (ESC). Endorsed by the European. European Heart Journal 2009;30(19):2369–413.

40. Calain P, Krause KH, Vaudaux P, et al. Early termination of a prospective, randomized trial comparing teicoplanin and flucloxacillin for treating severe

For peer review only

staphylococcal infections. The Journal of Infectious Diseases 1987;155(2):187–91.

41. Valente AM, Jain R, Scheurer M, et al. Frequency of infective endocarditis among infants and children with *Staphylococcus aureus* bacteremia. Pediatrics 2005;115(1):e15–9.

42. Ross AC, Toltzis P, O'Riordan MA, et al. Frequency and risk factors for deep focus of infection in children with *Staphylococcus aureus* bacteremia. The Pediatric Infectious Disease Journal 2008;27(5):396–9.

43. Johnson AP, Sharland M, Goodall CM, et al. Enhanced surveillance of methicillin-resistant *Staphylococcus aureus* (MRSA) bacteraemia in children in the UK and Ireland. Archives of Disease in Childhood 2010;95(10):781–5.

44. Le Saux N, Howard A, Barrowman NJ, Gaboury I, Sampson M, Moher D. Shorter courses of parenteral antibiotic therapy do not appear to influence response rates for children with acute hematogenous osteomyelitis: a systematic review. BMC infectious diseases 2002;2(16).

For peer review only

Table 1: Baseline characteristics of children with *Staphylococcus aureus* bacteraemia included in the study

Baseline variable	Pre-IDC group N=28 patients (%)	IDC group N=35 patients (%)	Combined N=63 patients (%)
Male	20 (71.4)	20 (57.1)	40 (63.5)
Female	8 (28.6)	15 (42.9)	23 (36.5)
Median age in years (IQR)	4.3 (0.2 – 9.4)	3.4 (0.2 – 12.2)	3.4 (0.2 – 10.7)
Neonates	6 (20)	9 (25.0)	15 (23.8)
Prematurity	4 (66.7)	9 (100.0)	13 (86.7)
Median age in days (IQR)	11 (7 – 47)	26 (23 – 38)	25 (14 – 47)
Median birth weight in grams (IQR)	1117 (630-3535)	820 (755-1120)	830 (710 – 1330)
Congenital heart disease	5 (17.9)	8 (22.9)	13 (20.6)
Chronic Pulmonary Disease	3 (10.7)	5 (14.3)	8 (12.7)
Liver Disease	0	1 (2.9)	1 (1.6)
Malignancy	7 (25.0)	9 (25.7)	16 (25.4)
Metastatic cancer	2 (7.1)	1 (2.9)	3 (4.8)
Neurological condition	4 (14.3)	13 (37.1)	17 (27.0)
Diabetes mellitus	0	1 (2.9)	1 (1.6)
Skin Condition	3 (10.7)	1 (2.9)	4 (6.4)
Atopic dermatitis	3 (100.0)		3 (75.0)
Immunosuppression	6 (21.4)	8 (22.9)	14 (22.2)
Corticosteroid therapy	2	3	5
Anti-neoplastic	5	5	10
Neutropenia	2	0	2
	**N=30 episodes (%)**	**N=36 episodes (%)**	**N=66 episodes (%)**
Mode of Acquisition			
Community-acquired	11 (36.7)	10 (27.8)	21 (31.8)
Healthcare-associated	9 (30.0)	7 (19.4)	16 (24.2)
Hospital-acquired	10 (33.3)	19 (52.8)	29 (43.9)
Duration of symptoms of bacteremia (hours)			
0-24	21 (70.0)	18 (50.0)	39 (59.1)
25-72	1 (3.3)	6 (16.7)	7 (10.6)
>72	7 (23.3)	10 (27.8)	17 (25.8)
Unknown	1 (3.3)	2 (5.6)	3 (4.6)
Organism			
MSSA	28 (93.3)	33 (91.7)	61 (92.4)
MRSA	2 (6.7)	3 (8.3)	5 (7.6)
C-Reactive Protein (nmol/L)	333 (114 – 890)	448 (181 – 1081)	390 (133 –1005)
White cell count (10^9/L)	8.8 (5.8 – 15.6)	10.1 (6.9 – 18.4)	9.6 (6.3 – 16.5)
Neutrophils (x 10^9/L)	5.4 (3.6 – 9)	6.3 (4.9-11.8)	5.7 (3.6 – 11.8)
Platelets (x 10^9/L)	277 (151 – 374)	213 (101 – 270)	224 (140 – 301)

IDC = infectious diseases consultation; IQR = interquartile range; MSSA = methicillin-susceptible *Staphylococcus aureus*; MRSA = methicillin-resistant *Staphylococcus aureus*

For peer review only

Table 2: Clinical features of *Staphylococcus aureus* bacteraemia in children included in the study

Focus of infection at time of bacteremia	Pre-IDC group N=30 episodes (%)	IDC group N=36 episodes (%)	Combined N=66 episodes (%)	P-value
Unknown focus	7 (23.3)	2 (5.6)	9 (13.6)	NS
Intravascular catheter	8 (26.7)	22 (61.3)	30 (45.5)	0.013
Culture confirmed	5 (62.5)	11 (50.0)	16 (53.3)	NS
Thrombophlebitis	0	2 (5.7)	2 (3.0)	NS
Bone/Joint infection	8 (26.7)	8 (22.2)	16 (24.2)	NS
Culture confirmed	4 (50.0)	2 (25.0)	6 (37.5)	NS
Lung	1 (3.3)	1 (2.8)	2 (3.0)	NS
Culture confirmed	0	1 (100.0)	1 (50)	NS
Skin & Soft tissue	7 (23.3)	9 (25.0)	16 (24.2)	NS
Culture confirmed	4 (57.1)	8 (88.9)	12 (75.0)	NS
Deep tissue abscess	2 (6.7)	1 (2.8)	3 (4.6)	NS
Culture confirmed	2 (100.0)	0	2 (66.7)	NS
Other focus	2 (6.7)	4 (11.1)	6 (9.1)	NS
Defervescence at 72 hours				
Yes	18 (60.0)	20 (55.6)	38 (57.6)	NS
No	11 (36.7)	14 (38.9)	25 (37.9)	
Unknown	1 (3.3)	2 (5.6)	3 (4.6)	

IDC = infectious diseases consultation; NS = non-significant

For peer review only

Table 3: Risk factors for *Staphylococcus aureus* bacteraemia in children included in the study

Risk factor for SAB	Pre-IDC group N=30 episodes (%)	IDC group N=36 episodes (%)	All children N=66 (episodes)	P-value
Age <1 year	11 (36.7)	14 (38.9)	25 (37.9)	NS
Underlying medical condition	16 (53.3)	24 (66.7)	40 (60.6)	NS
Duration in hospital in days (IQR) Prior to bacteremia*	11.5 (7.0-21.0)	19.0 (12.0-37.0)	16.0 (8.0-24.0)	NS
Prosthetic material	14 (46.7)	26 (72.2)	40 (60.6)	0.04
Intravascular line	13 (43.3)	23 (63.9)	36 (54.6)	NS
Endotracheal tube	0	4	4	NS
Other	1	6	8	NS
Corticosteroid therapy	2 (6.7)	3 (8.3)	5 (7.6)	NS
Surgery within previous 30 days	4 (13.3)	4 (11.1)	8 (12.1)	NS

IDC=infectious disease consultation; IQR=interquartile range; NS = non-significant; SAB=*Staphylococcus aureus* bacteremia
*Hospital-acquired infection only

For peer review only

Table 4: Comparison of the management and outcome of *Staphylococcus aureus* bacteraemia in children, pre- and post-introduction of an infectious disease consult service.

**Quality indicator**	**Pre-IDC group<br>N=30 episodes (%)**	**IDC group<br>N=36 episodes (%)**	**P-Value**
Median time to infectious diseases review in days (IQR)	N=4<br>3.5 (0.5-21.5)	N=34<br>2.0 (1.0-4.0)	NS
Repeat blood culture performed	26 (86.7)	32 (88.9)	NS
Time to repeat blood culture<br>0-48 hours<br>48-96 hours<br>> 96 hours	 20<br>8<br>1	 18<br>13<br>1	 NS<br>NS<br>NS
Repeat blood culture positive<br>0-48 hours<br>48-96 hours<br>> 96 hours	6<br>3<br>0	6<br>4<br>0	NS<br>NS<br>NS
Echocardiogram performed<br>Yes<br>No	 10 (33.3)<br>20 (66.7)	 29 (80.6)<br>7 (19.4)	 0.0001
Beta-Lactam therapy<br>MSSA	27 (90.0)<br>25 (92.6)	34 (94.4)<br>33 (97.1)	NS
Removable focus of infection<br>Focus removed<br>Median time to removal in days (IQR)	7 (23.3)<br>6 (85.7)<br>2.0 (2.0-2.0)	22 (43.9)<br>21 (95.5)<br>3.0 (1.0-18.0)	0.003<br>NS<br>NS
Median time to appropriate antibiotics in days (IQR)	0.0 (0)	0.0 (0)	NS
Median duration of IV antibiotics in days (IQR)	13.5 (7.0-21.0)	18.0 (15.0-29.0)	0.035
Median duration of IV and/or oral antibiotics in days (IQR)	20.5 (16.0-42)	19.0 (15.0 – 29.5)	NS
**Complicated infection**	**N=15 episodes<br>Days (IQR)**	**N=19 episodes<br>Days (IQR)**	
Median duration of IV antibiotics<br>Median duration of IV or oral antibiotics<br>Median duration of IV antibiotics if repeat blood culture positive<br>Met standard recommendation 28 days IV antibiotics (%)	14.0 (6.0-21.0)<br>19.0 (17.0-43.0)<br>13.0 (6.0 – 14.0)<br>2 (13.3)	22 (15.0-39.0)<br>27.0 (16.0-39.0)<br>19.0 (15.0 – 27.0)<br>8 (42.1)	0.02<br>NS<br>0.007<br>NS
**Uncomplicated infection**	**N=15 episodes<br>Days (IQR)**	**N=17 episodes<br>Days (IQR)**	
Median duration of IV antibiotics<br>Median duration of IV or oral antibiotics (IQR)<br>Met standard	13.0 (7.0 – 22.0)<br>22.0 (14.0-32.0)	15.0 (14.0-21.0)<br>18.0 (14.0-29.0)	NS<br>NS

For peer review only

recommendation of 14 days (%)	7 (46.7)	13 (68.4)	NS
**Outcomes**	**N=30 episodes Days (IQR)**	**N=36 episodes Days (IQR)**	
Median duration of hospital admission<br>Total<br>Complicated<br>Uncomplicated	 <br>14.0 (6.0-42.0)<br>20.0 (6.0 – 49.0)<br>7.0 (3.0-22.0)	 <br>16.5 (7.5-58)<br>25.0 (8.0-88.0)<br>11.0 (6.0-36.0)	 <br>NS<br>NS<br>NS
SAB recorded in discharge summary	24 (80.0)	29 (82.9)	NS
Secondary infection detected	1	3	NS
Outcomes 30-day post-SAB<br>Death<br>Recurrence	 0<br>0	 3<br>0	 NS<br>NS
Outcomes 30-90 days post SAB<br>Death<br>Recurrence	 2<br>2	 0<br>1	 NS<br>NS

IDC = infectious diseases consultation; IQR = Interquartile range; IV = intravenous; NS = non-significant; SAB = *Staphylococcus aureus* bacteremia; Mortality was analysed per patient (only the first episode was analysed).

For peer review only

Figure legend

Figure 1. Study schema of paediatric patients with *S. aureus* bacteraemia

Title

Impact of infectious diseases consultation on the management of *S. aureus* bacteraemiabactaeremia in children

Authors

Rebecca B. Saunderson,[1] Theodore Gouliouris,[1,2,3] Edward J. Cartwright,[1,3] Emma J. Nickerson,[4] Sani H. Aliyu,[2,4] D. Roddy O'Donnell,[5] Wilf Kelsall,[5] D. Limmathurotsakul,[7] Sharon J. Peacock,[1,2,3,6] M. Estée Török[1,2,3]

Affiliations

1. Department of Medicine, University of Cambridge, Cambridge, United Kingdom

2. Department of Microbiology, Cambridge University Hospitals NHS Foundation Trust, Cambridge, United Kingdom

3. Public Health England, Clinical Microbiology and Public Health Laboratory, Cambridge, United Kingdom

4. Department of Infectious Diseases, Cambridge University Hospitals NHS Foundation Trust, Cambridge, United Kingdom

5. Department of Paediatrics, Cambridge University Hospitals NHS Foundation Trust, Cambridge, United Kingdom

6. Wellcome Trust Sanger Institute, Hinxton, United Kingdom

7. Mahidol Oxford Research Unit, Mahidol University, Bangkok, Thailand

Correspondence:

Dr M. Estée Török

University of Cambridge, Department of Medicine, Box 157, Addenbrooke's Hospital, Hills Road, Cambridge CB2 0QQ, United Kingdom

Telephone: +44 (0) 1223 336 845

Facsimile: +44 (0) 1223 336 846

Email: et317@medschl.cam.ac.uk

Key words

Staphylococcus aureus bacteraemia; infectious diseases consultation; management; outcome; paediatric

Word count

Abstract = 260 words; Main text = 3,6053214; Tables = 4; Figures=1

For peer review only

ABSTRACT

Objectives

Infectious diseases consultation (IDC) in adults with *Staphylococcus aureus* bacteraemia (SAB) has been shown to improve management and outcome. The aim of this study was to evaluate the impact of IDC on the management of SAB in children.

Study design

Observational cohort study of children with SAB.

Setting

Cambridge University Hospitals NHS Foundation Trust, a large acute NHS Trust in the United Kingdom.

Participants

All children with SAB admitted to the Cambridge University Hospitals NHS Foundation Trust between 16 July 2006 and 31 December 2012.

Methods

Children with SAB between 2006 and 31 October 2009 were managed by routine clinical care (pre-IDC group) and data were collected retrospectively by case notes review. An IDC service for SAB was introduced in November 2009. All children with SAB were reviewed regularly and data were collected prospectively (IDC group) until 31 December 2012. Baseline characteristics, quality metrics, and outcome were compared between the pre-IDC group and IDC group.

Results

There were 66 episodes of SAB in 63 children –; 28 patients (30 episodes) in the pre-IDC group, and 35 patients (36 episodes) in the IDC group. The median age was 3.4 years (IQR 0.2 – 10.7 years). Patients in the IDC group were more likely to have echocardiography performed, a removable focus of infection identified, and to receive

For peer review only

a longer course of intravenous antimicrobial therapy. There were no differences in total duration of antibiotic therapy, duration of hospital admission, or outcome at 30 or 90 days following onset of SABbacteraemia.

Conclusions

IDC resulted in improvements in the investigation and management of SAB in children.

For peer review only

ARTICLE SUMMARY

Strengths and limitations of the study

- This is the first study to examine the impact of introduction of infectious diseases consultation (IDC) on the management of *S. aureus* bacteraemia (SAB) in children.
- We found that IDC was associated with an improvement in investigation and management of SAB, but there was no difference in mortality between the pre-IDC and IDC groups.
- The main limitation of the study was the size of the study population, which may explain the lack of mortality benefit.
- The study was conducted in a tertiary referral centre, where clinical management is likely to have been good prior to introduction of the IDC, and may not be generalizable to other settings.

For peer review only

MAIN TEXT

Introduction

Staphylococcus aureus bacteraemia (SAB) is a serious infection that leads to significant morbidity and mortality in adults and children.[1,2] *Staphylococcus aureus* (*S. aureus*) causes significant disease in the paediatric population, occurring in 1.5% of all neonatal intensive care unit (ICU) admissions[3], and 6 per 100,000 children older than 1 year of age.[4] In neonates, SAB is almost always hospital-acquired, and is frequently due to intravascular catheter (IVC) associated infections.[3–6] The majority of non-neonatal cases of SAB are community-acquired; those that are hospital-acquired infections are usually IVC-associated.[7,8]

Identified risk factors for the development of SAB in the paediatric population include having a pre-existing medical condition, prolonged hospitalization, the presence of an IVC, and HIV infection.[1,3–6,9,10] Mortality from SAB in the adult population is about 30% .%.[11] Mortality rates in the paediatric population tend to be lower, but can be up to 15% in neonates and/or children with co-morbidities.[1,5,9,12,13] Given that SAB causes a substantial burden of disease in the paediatric population, strategies to improve management, prevent the complications of SAB, and reduce mortality are a clinical priority.

The impact of infectious disease consultation (IDC) in adults with SAB has been extensively studied . .[14–24] IDC has been associated with improved adherence to guidelines~~evidence-based practice~~, including appropriate and targeted investigation, optimal duration of antibiotic therapy, and a reduction in complicated infection, morbidity and mortality.[14,16,17,22,23,25–28] In contrast, the impact of an IDC on the

For peer review only - http://bmjopen.bmj.com/site/about/guidelines.xhtml

management and outcomes of SAB in children has not previously been evaluated. The aim of this study was to determine the effects of routine IDC on the investigation, management, and outcome of children with SAB.

Materials and methods

Study setting and participants

Cambridge University Hospitals National Health Service Foundation Trust (CUH) is a tertiary referral centre for paediatrics in the East of England. The paediatric service has a 22-bed medical and surgical ward, a 17-bed paediatric haematology and oncology ward, an 11-bed paediatric ICU and high dependency unit (both caring for children aged from 0 to 16 years), and a 12-bed surgical and medicalmedicine ward for children aged up to 3 years. The Rosie Hospital, the on-site mother and baby hospital, has a 17-cot Neonatal ICU and a 10-cot Special Care Baby Unit.

Study design

We conducted an observational cohort study of all children with SAB admitted to CUH betweenfrom 16 July 2006 anduntil the 31 December 2012. In November 2009 an IDC service for all patients with SAB was established at CUH. IDC comprised an initial clinical review, followed by weekly follow-up until the time of hospital discharge. Data were collected from 2006 to 2009 by a retrospective review of the medical records, and prospectively thereafter during the IDC service. Demographic, clinical and microbiology information were collected using a case record form, and entered into an electronic database. Patients with contaminated blood cultures that were considered to be contaminants (afebrile with no clinical evidence of infection) or withor polymicrobial blood cultures were excluded from the analysis.

For peer review only

Microbiological investigation

Blood cultures were collected and incubated at 37°C for 5 days using BacT/Alert 3D system (bioMérieux, Basingstoke, UK). Blood cultures that flagged positive were examined by microscopy and presumptively identified as *S. aureus* using a thermostable nuclease test.[29] Colonies of *S. aureus* were identified by routine methods after a further overnight incubation. Identification of *S. aureus* was performed using a commercial latex agglutination test (Staphaurex, Remel, Lenexa, USA) until 2011 and then using matrix-assisted laser desorption ionization time-of-flight mass spectroscopy (Bruker Daltonik, Bremen, Germany). Antibiotic susceptibilities were determined using disc diffusion testing, according to British Society for Antimicrobial Chemotherapy standards.[30] Throughout the study period a clinicalmedical microbiologist provided telephone advice to the clinical team for all patients with SAB, and attended weekly ward rounds on the paediatric oncology ward and paediatric ICU.

Study procedures

Prior to November 2009 all patients with *S. aureus* bacteraemia were managed by their primary clinical care team, with telephone advice from the microbiologists. From November 2009, all patients with SAB were reviewed by an infectious diseases Specialist Registrar or Consultant,a member of the IDC team following presumptive identification of *S. aureus* in the blood cultures.culture. The assessment included clinical history to determine symptoms of infection, and physical examination to determine possible foci of infection. Patients underwent clinical review daily by their primary care team and at least weekly by the IDC team during their inpatient stay.

For peer review only

Demographic, clinical and microbiology data were collected using a standard case record form, and entered into an electronic database.

An IVC was considered to be the focus of infection if there was evidence of inflammation at the catheter exit site and/or a vascular catheter tip culture positive for *S. aureus,* without clinical evidence of another source of bacteraemia.[31] Thrombophlebitis was diagnosed when there was clinical evidence of infection and inflammation along a blood vessel or when ultrasound or other imaging confirmed the presence of intravascular thrombosis in the setting of suspected infection. Bone and joint infections were defined according to United States Centers for Disease Control and Prevention criteria.[32] The lung was considered to be the source of infection when there was clinical, radiological and/or microbiological evidence of pulmonary infection. Soft tissue infection was considered to be the source of the bacteraemia if the clinical signs of a known or suspected soft tissue infection pre-dated or were present at the time of bacteremia.[14] A deep tissue abscess was defined by radiological imaging criteria. Infective endocarditis (IE) was diagnosed according to the modified Duke criteria.[33,34]

A SAB episode was defined as being greater than or equal to 14 days from a previous episode, in the absence of persistent bacteraemia or focus of infection. A secondary site of infection was defined as a site of infection separate from the primary site of infection that was not present at the time of the initial examination. Healthcare-associated bloodstream infection was defined according to previously published criteria.[35] Hospital-acquired infection was defined according to United States Centers for Disease Control and Prevention ..[32] Community-acquired infections were defined

For peer review only - http://bmjopen.bmj.com/site/about/guidelines.xhtml

as those patients with a positive blood culture taken at or within 48-hours of admission who did not meet criteria for healthcare-associated bloodstream infection.[35] Patients were classified as having uncomplicated SAB if blood cultures were negative two to four days after the initial blood culture was positive, if they had defervesced at 72 hours, if there was no evidence of metastatic disease or endocarditis, or if they had a catheter related infection.[36]

Appropriate antimicrobial therapy was defined as therapy to which the isolate was determined to be susceptible by antimicrobial disc susceptibility testing. The duration of therapy was the length of time that a patient received antibiotics to which the isolate was susceptible. An underlying medical condition was defined as any chronic medical condition that was present at the time of bacteraemia. Serum C-reactive protein (CRP), blood, white cell counts, and platelet countsplatelets were measured on the day of, or within 48 hours post -bacteraemia. Duration of hospital admission and outcome at 30 and 90 days post-bacteraemia were recorded for all patients.

Treatment recommendations

Antimicrobial treatment recommendations were provided for all children with SAB, based on existing evidence on the management of SAB in adults.[14,37–40] These included removal of a removable focus of infection,[14] performing repeat blood cultures at 48 to 96 hours,[37] performing a transthoracic echocardiogram, performing radiological imaging of suspected deep foci of infection, treating uncomplicated infection with 14 days of intravenous (IV) antibiotics,[38] treating complicated infections with a minimum of 28 days of IV antibiotics[39], and using beta-lactam therapy as the mainstay of treatment for methicillin-susceptible *S. aureus*..[40]

For peer review only

Statistical analysis

Data were analyzed using STATA version 12 (StataCorp, College Station, Texas, USA). Categorical variables were analyzed using the Fisher's exact test and reported as the number and percent. Continuous variables were compared using the Mann Whitney U test and reported as the median and interquartile range. Mortality was analysed per patient (i.e. only the first bacteraemia episode was analysed).

Ethics statement

Written informed consent from participants was not required as the study was conducted as a service evaluation. The study protocol received approval fromwas approved by the University of Cambridge Human Biology Research Ethics Committee, and the CUH Research and Development Department.

Results

Patient characteristics

Between July 2006 to December 2012, 71 children had one or more blood cultures that were positive for *S. aureus.* Sixty-three children (66 episodes) were included in the study. Five children (six episodes) were excluded because of polymicrobial bacteraemia andinfection. Three patients (three episodes) were excluded becausewhen the cultures were considered to be contaminants.culture was interpreted as representing contamination following clinical assessment. Thus, 28 patients (30 episodes) were included in the pre-intervention (pre-IDC) group, and 35 patients (36 episodes) in the intervention (IDC) group. The study schema is summarized in Figure 1. Four of 30 episodes (13patients (14.3%) received an IDC before the service was

For peer review only

implemented in 2009, and 34 of 36 episodespatients (94.4%) received an IDC after the service was implemented in 2009.

The baseline characteristics of the two groupsstudy population are presented in Table 1. These were similar (Table 1).apart from a higher serum C-reactive protein in the IDC group (448 nmol/L versus 333nmol/L, P=0.047). The clinical features for SAB were likewise similar, apart from an increased proportion of IVC-relatedassociated infections in the IDC group (61.3% versus 26.7%, P≤=0.01013) (Table 2). A higher proportion of patients had an unidentified focus of infection in the pre-IDC group compared with the IDC group (23.3% versus 5.6%, P=0.07).068). The risk factors for SAB were also similar apart from an increased frequency of prosthetic materialIVC in the IDC group (72.263.9% versus 46.743.3%, P=0.04137) (Table 3).

Clinical management

A service evaluation of the IDC service was conducted, the results of which are summarized in Table 4. In the IDC group, 34/36 episodes had an infectious diseases review, with a median time to review of two days (range 1 to 4 days). Patients in the IDC group were more likely to have a transthoracic echocardiographyechocardiogram performed (80.6% versus 33.3%, P≤=0.010001). They were also more likely to have a removable focus of infection identified (43.9% versus 23.3%, P≤=0.01003), although there was no difference between the two groups in the likelihood of removal, or the time to removal. In the IDC group two patients did not have their IVC removed, despite the recommendation to do so, because of concerns about difficulty in re-establishing vascular access. There was no difference in the number of repeat blood cultures performed between groups.

For peer review only

Antimicrobial therapy

There was no difference between the two groups in the time taken to initiate appropriate antimicrobial therapy. Patients in the IDC group were more likely to receive a longer duration of IV antimicrobial therapy (18 days versus 13.5 days, P=0.04~~035~~), although there was no difference in total duration of therapy (IV and oral) between the two groups. In patients with complicated SAB, the duration of IV antibiotic therapy was longer in the IDC group (22 days versus 14 days, P=0.02) although there was no difference in total duration of antibiotic therapy (IV and oral) between the two groups. Patients in the IDC group were more likely to receive a longer duration of IV therapy if their repeat blood culture result was positive (P<~~=~~0.01~~007~~). In patients with uncomplicated SAB there was no difference between groups in the duration of IV antibiotics, or the total duration of antibiotic therapy. In terms of compliance with recommended standards for duration of therapy, patients in the IDC group were more likely to meet these standards compared with patients in the non-IDC group, both for complicated SAB (42.1% versus 13.3%, P=0.13) and uncomplicated SAB (68.4% versus 46.7%, P=0.14). There was~~were~~ no difference~~differences~~ in the proportion of patients receiving beta-lactam therapy for MSSA bacteraemia between the two groups.~~.~~

For peer review only

Outcome of SAB

The duration of hospital admission was similar in the pre-IDC and IDC groups, and in those with uncomplicated and complicated SAB (Table 4). SAB was recorded in the discharge summary in the majority of patients in both groups. Four secondary infections were diagnosed, three in the IDC group and one in the pre-IDC group. In

the IDC group, the secondary infections were tricuspid valve endocarditis (in a very low birth weight neonate with patent ductus arteriosus)~~,~~ pneumonia, and osteomyelitis, respectively. One child in the non-IDC group developed osteomyelitis. There were three cases of recurrent bacteraemia within 90 days – ~~, of which~~ two ~~were~~ in the pre-IDC group and one ~~was~~ in the IDC group. Three children died within 30 days of SAB, all in the IDC group, giving an overall 30-day mortality rate of 4.8%. One death occurred in a child with metastatic cancer and was not attributed to the SAB. The other two deaths were deemed attributable to SAB, as blood cultures were positive at the time of death. One patient was a neonate and died prior to IDC, and the second case had cerebellar atrophy, developmental delay and was receiving total parenteral nutrition. The 90-day ~~overall~~ mortality rate was 7.9%. Two patients died between 30 and 90 days post-SAB, both in the pre-IDC group. One patient had metastatic cancer, and the other had complex congenital heart disease.

Discussion

To our knowledge this is the first study to systematically examine the impact of introduction of an IDC service on the management of SAB in children. We compared the clinical features, management, and outcomes of all children ~~with~~ presenting with SAB to our hospital between July 2006 and December 2012, before and after introduction of an IDC service. The main findings of the study were that patients in the IDC group were more likely to have echocardiography performed, a removable focus of infection identified, and to receive a longer course of IV antibiotic therapy. These findings concur with those from previous studies of IDC conducted in adults with SAB, and reflect current best practice.

For peer review only

Follow-up blood cultures have been recommended in adults, as prolonged bacteraemia is a predictor of complicated infection and poorer outcome in SAB.[37] As a result, prolonged IV antibiotic therapy is recommended in patients with positive repeat blood cultures. We found that children who had a positive repeat blood culture were more likely to receive a longer course of IV antibiotics if they were in the IDC group.

Echocardiography was performed in a higher proportion of children in the IDC group compared with the pre-IDC group. The rates of IE in children with SAB are reported to be between 0 and ~~-~~20%, which is similar to rates reported in the adult population.[1,3,6,8,12,41–43] An American study by Valente and colleagues diagnosed IE in 20% of children with SAB (~12% of whom had confirmed IE)).[41] Children with underlying congenital heart disease had a higher prevalence of confirmed or probable IE compared to those who had structurally normal hearts (53% versus 3%) and patients with definite IE had multiple positive blood cultures. Mortality was higher in patients with endocarditis compared to those without (40% versus 12%). Another study from South Africa reported an IE rate of 11% in children with SAB.[12] Risk factors for the development of IE in children include congenital heart disease, a central IVC, and persistently positive blood cultures after 24 hours.[41] ~~-~~,[42] In the United Kingdom, there are no published guidelines on the use of echocardiography in children with SAB. The Infectious Diseases Society of America guidelines for MRSA bacteraemia recommend performing echocardiography in children with congenital heart disease, those with bacteraemia duration greater than two days, or those with other clinical findings suggestive of endocarditis.[36] In our~~this~~ study the one child who developed tricuspid valve endocarditis was a very low birth weight

For peer review only

premature neonate with a patent ductus arteriosus, an IVC-related infection, and persistent bacteremia. Our findings concur with these guidelines, and support the use of a risk-based strategy for the use of echocardiography in children with SAB.

We also found that a higher proportion of children had an IVC-related infection, and/or a removable focus of infection in the IDC group compared with the pre-IDC group, although neither of these differences were statistically significant. It is possible that IVC was used more during the IDC period compared with the pre-IDC period. Although removable foci of infection were more frequently identified and removed in the IDC group compared to the pre-IDC group, the median time to removal was slightly longer (3 versus 2 days). In some cases this was related to practical difficulties in removing the focus, such as re-establishing vascular access in neonates.. Conversely, there were fewer patients with an unidentified focus of infection in the IDC group compared with the pre-IDC group. These findings indicate that the introduction of specialist IDC serviceinfectious diseases review improved the rate of diagnosis of the focus of infection in children with SAB, suggesting that the consult service was beneficial.

The frequency of MRSA bacteraemia was only 7.6% in our study cohort. Possible explanations for this are that MRSA bacteraemia is less common in children than in adults , and that MRSA bacteraemia rates have significantly declined in the United Kingdom since 2001 . By contrast, in other countries such as the United States, the incidence of MRSA bloodstream infections have been higher than in the UK , but have recently declined . In a large retrospective study of over 57,000 hospitalised children with *S. aureus* infections, 51% had MRSA and 61% had MRSA skin and soft

For peer review only - http://bmjopen.bmj.com/site/about/guidelines.xhtml

tissue infections. The incidence of skin and soft tissue infections, pneumonia, osteomyelitis and bacteraemia increased over time but overall mortality was low (1%) . Thus the findings of our study may not be generalizable to other settings where the epidemiology and outcomes of MRSA bacteraemia are different.

In terms of duration of antimicrobial therapy, we found that patients in the IDC group received longer courses of IV antibiotics in complicated infection, compared with patients in the pre-IDC group. This concurscorresponds with findings from studies in adults with SAB, and suggests that specialist infectious diseases review may be beneficial in ensuring that clinical management recommendations, such as the length of IV antimicrobial therapy, are applied. There were,. However, no differences in morbidity or mortality observed between the pre-IDC and IDC groups. were found. The most likely explanation for this was the small study population, combined with a low (63 patients in total and only 35 in the IDC group). The mortality rate, which meant that of SAB in children is low so a large number of patients would be required to demonstrate even in small difference in mortality. Furthermore the overall duration of antibiotic therapy was similar in the two groups., although the IDC group did receive longer courses of IV antibiotics. It may be that total duration of antibiotic therapy is more important than the route of administration, provided that adequate concentrations arecan be achieved in the bloodstream.blood. Indeed studies to examine this very question, comparing short versus long courses of IV antibiotics in SAB*S. aureus* bacteraemia in adults are ongoing., and should also be conducted in the paediatric population. Finally, although a removable focus of infection was identified more frequently in the IDC group, the likelihood offor removal and the time to removal did not differ; this may also explain the lack of difference in outcome

between the two groups.

We acknowledge several limitations to our study. The study population was small and the differences in diagnosis and management that we observed in the two groups did not translate into differences in outcome, for reasonsas discussed above. The retrospective data collection during the pre-IDC period (2006 to 2009) carries a risk of incomplete recording of data and potential bias. There were, however, no differences in baseline characteristics between the two groups in terms of age, gender, underlying co-morbidities or focus of infection. The only exception was a higher frequency of IVC-related infections in the IDC group, which may have been under-recordedreported in the pre-IDC period and / /or diagnosed more frequently in the IDC period.

In conclusion, we found that introduction of IDC for paediatric SAB resulted in improvements in management and a more consistent approach to care across the paediatric service. These findings concur with those of previous studies of IDC in adults with SAB. Our findings and support the use of a targeted approach to echocardiography in SAB in children, particularly for patients with risk factors for complicated disease. Despite improvements in the investigation and clinical management, we did not find any differences in the development of secondary infections, recurrent bacteremia, or death between the two groups. The most likely explanation for this is the small study population and larger prospective studies are required validate our findings and to determine the optimal strategies for investigation and management of paediatric SAB.

For peer review only

Funding

This work was supported by grants from the United Kingdom Clinical Research Collaboration (UKCRC) Translational Infection Research Initiative (TIRI); the Medical Research Council (G1000803), with contributions from the Biotechnology and Biological Sciences Research Council, the National Institute for Health Research (NIHR) on behalf of the United Kingdom Department of Health, and the Chief Scientist of the Scottish Government Health Directorate; the Public Health England; and the NIHR Cambridge Biomedical Research Centre. MET is a Clinician Scientist Fellow funded by the Academy of Medical Sciences at the Health Foundation.

Competing interests statement

All authors have no conflicts of interest to declare.

Author contributions

RBS was part of the infectious diseases consultation service, collected the data, performed the data analysis, and wrote the first draft of the manuscript. TG was part of the infectious diseases consultation service, collected the data, and contributed to writing the manuscript. EJC collected the data, and contributed to the data analysis writing the manuscript. EJN was part of the infectious diseases consultation service, collected the data, and contributed to writing the manuscript. SHA was part of the infectious diseases consultation service, collected the data, and contributed to writing the manuscript. DRO'D was part of the clinical care team, and contributed to writing the manuscript. WK was part of the clinical care team and contributed to writing the manuscript. SJP conceived and supervised the study, and contributed to writing the manuscript. MET conceived and supervised the study, led the infectious diseases

For peer review only

consultation service, collected the data, contributed to data analysis, and writing the manuscript. All authors approved the final manuscript.

References

1. Burke RE, Halpern MS, Baron EJ, Gutierrez K. Pediatric and neonatal *Staphylococcus aureus* bacteremia: epidemiology, risk factors, and outcome. Infection Control and Hospital Epidemiology 2009;30(7):636–44.

2. Mylotte JM, Tayara A. *Staphylococcus aureus* bacteremia: predictors of 30-day mortality in a large cohort. Clinical Infectious Diseases 2000;31(5):1170–4.

3. Healy CM, Palazzi DL, Edwards MS, Campbell JR, Baker CJ. Features of invasive staphylococcal disease in neonates. Pediatrics 2004;114(4):953–61.

4. Frederiksen MS, Espersen F, Frimodt-Møller N, et al. Changing epidemiology of pediatric *Staphylococcus aureus* bacteremia in Denmark from 1971 through 2000. The Pediatric Infectious Disease Journal 2007;26(5):398–405.

5. Hakim H, Mylotte JM, Faden H. Morbidity and mortality of Staphylococcal bacteremia in children. American Journal of Infection Control 2007;35(2):102–5.

6. Denniston S, Riordan FAI. *Staphylococcus aureus* bacteraemia in children and neonates: a 10 year retrospective review. The Journal of Infection 2006;53(6):387–93.

7. Hill PC, Wong CGS, Voss LM, et al. Prospective study of 125 cases of *Staphylococcus aureus* bacteraemia in children in New Zealand. The Pediatric Infectious Disease Journal 2001;20(9):868–73.

8. Gray J, O'Donoghue B. Bacteraemia with meticillin-susceptible *Staphylococcus aureus* in an English children's hospital. The Journal of Hospital Infection 2011;78(2):158–9.

9. Groome MJ, Albrich WC, Wadula J, Khoosal M, Madhi SA. Community-onset *Staphylococcus aureus* bacteraemia in hospitalised African children: high incidence in HIV-infected children and high prevalence of multidrug resistance. Paediatrics and International Child Health 2012;32(3):140–6.

10. Suryati BA, Watson M. *Staphylococcus aureus* bacteraemia in children: a 5-year retrospective review. Journal of Paediatrics and Child Health 2002;38(3):290–4.

11. Wyllie DH, Crook DW, Peto TEA. Mortality after *Staphylococcus aureus* bacteraemia in two hospitals in Oxfordshire, 1997-2003: cohort study. BMJ Online First 2006;333(7562):281.

For peer review only

12. Friendland IR, du Plessis J, Cilliers A. Cardiac complications in children with *Staphylococcus aureus* bacteremia. The Journal of Pediatrics 1995;127(5):476–8.

13. Carrillo-Marquez M a, Hulten KG, Mason EO, Kaplan SL. Clinical and molecular epidemiology of *Staphylococcus aureus* catheter-related bacteremia in children. The Pediatric Infectious Disease Journal 2010;29(5):410–4.

14. Fowler VG, Sanders LL, Sexton DJ, et al. Outcome of *Staphylococcus aureus* bacteremia according to compliance with recommendations of infectious diseases specialists: experience with 244 patients. Clinical Infectious Diseases 1998;27(3):478–86.

15. Lillie P, Moss P, Thaker H, et al. Development, impact and outcomes of the Hull Bacteraemia Service. QJM 2008;101(11):889–98.

16. Nagao M, Iinuma Y, Saito T, et al. Close cooperation between infectious disease physicians and attending physicians can result in better management and outcome for patients with *Staphylococcus aureus* bacteraemia. Clinical Microbiology and Infection 2010;16(12):1783–8.

17. Robinson JO, Pozzi-Langhi S, Phillips M, et al. Formal infectious diseases consultation is associated with decreased mortality in *Staphylococcus aureus* bacteraemia. European Journal of Clinical Microbiology & Infectious Diseases 2012;31(9):2421–8.

18. Jenkins TC, Price CS, Sabel AL, Mehler PS, Burman WJ. Impact of routine infectious diseases service consultation on the evaluation, management, and outcomes of *Staphylococcus aureus* bacteremia. Clinical Infectious Diseases 2008;46(7):1000–8.

19. Choi S-H, Cho SY, Park J-H, Chung J-W. Impact of infectious-disease specialist consultations on outcomes of *Staphylococcus aureus* bacteremia in a hospital with a low volume of patients with S. aureus bacteremia. The Journal of Infection 2011;62(2):181–5.

20. Kaech C, Elzi L, Sendi P, et al. Course and outcome of *Staphylococcus aureus* bacteraemia: a retrospective analysis of 308 episodes in a Swiss tertiary-care centre. Clinical Microbiology and Infection 2006;12(4):345–52.

21. Honda H, Krauss MJ, Jones JC, Olsen M a, Warren DK. The value of infectious diseases consultation in *Staphylococcus aureus* bacteremia. The American Journal of Medicine 2010;123(7):631–7.

22. Rieg S, Peyerl-Hoffmann G, de With K, et al. Mortality of S. aureus bacteremia and infectious diseases specialist consultation--a study of 521 patients in Germany. The Journal of Infection 2009;59(4):232–9.

23. Jenkins TC, Price CS, Sabel AL, Mehler PS, Burman WJ. Impact of routine infectious diseases service consultation on the evaluation, management, and

For peer review only

outcomes of *Staphylococcus aureus* bacteremia. Clinical Infectious Diseases 2008;46:1000–8.

24. De La Blanchardière A, Boutemy J, Thibon P, Michon J, Verdon R, Cattoir V. Clinical benefit of infectious diseases consultation: a monocentric prospective cohort study. Infection 2012;40(5):501–7.

25. Forsblom E, Ruotsalainen E, Ollgren J, Järvinen A. Telephone Consultation Cannot Replace Bedside Infectious Disease Consultation in the Management of *Staphylococcus aureus* Bacteremia. Clinical Infectious Diseases 2013;56(4):527–35.

26. Tissot F, Calandra T, Prod'hom G, et al. Impact of infectious diseases consultation on outcome of methicillin-resistant *Staphylococcus aureus* bacteraemia in a tertiar centre: a ten-year experience. 21st ECCMID/27th ICC 2010;:S420–421.

27. Minton J, Clayton J, Sandoe J, Mc Gann H, Wilcox M. Improving early management of bloodstream infection: a quality improvement project. BMJ 2008;336(7641):440–3.

28. Lundberg J, Nettleman MD, Costigan M, Bentler S, Dawson J, Wenzel RP. *Staphylococcus aureus* bacteraemia: The cost effectiveness of long-term therapy associated with infectious diseases consultation. Clinical Performance and Quality in Health Care 1998;6:9–11.

29. Enoch DA, Cooke FJ, Guha S, Brown NM. Thermostable nuclease: a study of clinical impact. The Journal of Antimicrobial Chemotherapy 2008;61(3):754–5.

30. BSAC. BSAC Methods for Antimicrobial Susceptibility Testing, Version 12 May 2013. http://bsac.org.uk/wp-content/uploads/2012/02/Version-12-Apr-2013_final.pdf.

31. Libman H, Arbeit R. Complications associated with *Staphylococcus aureus* bacteraemia. Archives of Internal Medicine 1984;144(3):541–5.

32. Appendix S, January F. CDC / NHSN Surveillance Definition of Healthcare-Associated Infection and Criteria for Specific Types of Infections in the Acute Care Setting. 2012;(January).

33. Li JS, Sexton DJ, Mick N, et al. Proposed modifications to the Duke criteria for the diagnosis of infective endocarditis. Clinical Infectious Diseases 2000;30(4):633–8.

34. Durack DT, Lukes AS, Bright DK. New Criteria for Diagnosis of Infective Endocarditis□: Utilization of Specific Echocardiographic Findings. The American Journal of Medicine 1994;96:200–9.

For peer review only

35. Friedman ND, Kaye KS, Stout JE, et al. Health Care–Associated Bloodstream Infections in Adults: A Reason To Change the Accepted Definition of Community-Acquired Infections. Annals of Internal Medicine 2002;10(137):791–7.

36. Liu C, Bayer A, Cosgrove SE, et al. Clinical practice guidelines by the Infectious Diseases Society of America for the treatment of methicillin-resistant *Staphylococcus aureus* infections in adults and children. Clinical Infectious Diseases 2011;52(3):e18–55.

37. Fowler VG, Olsen MK, Corey GR, et al. Clinical identifiers of complicated *Staphylococcus aureus* bacteraemia. Archives of Internal Medicine 2003;163:2066–72.

38. Mermel LA, Allon M, Bouza E, et al. Clinical practice guidelines for the diagnosis and management of intravascular catheter-related infection: 2009 Update by the Infectious Diseases Society of America. Clinical Infectious Diseases 2009;49(1):1–45.

39. Habib G, Hoen B, Tornos P, et al. Guidelines on the prevention, diagnosis, and treatment of infective endocarditis (new version 2009): the Task Force on the Prevention, Diagnosis, and Treatment of Infective Endocarditis of the European Society of Cardiology (ESC). Endorsed by the European. European Heart Journal 2009;30(19):2369–413.

40. Calain P, Krause KH, Vaudaux P, et al. Early termination of a prospective, randomized trial comparing teicoplanin and flucloxacillin for treating severe staphylococcal infections. The Journal of Infectious Diseases 1987;155(2):187–91.

41. Valente AM, Jain R, Scheurer M, et al. Frequency of infective endocarditis among infants and children with *Staphylococcus aureus* bacteremia. Pediatrics 2005;115(1):e15–9.

42. Ross AC, Toltzis P, O'Riordan MA, et al. Frequency and risk factors for deep focus of infection in children with *Staphylococcus aureus* bacteremia. The Pediatric Infectious Disease Journal 2008;27(5):396–9.

43. Johnson AP, Sharland M, Goodall CM, et al. Enhanced surveillance of methicillin-resistant *Staphylococcus aureus* (MRSA) bacteraemia in children in the UK and Ireland. Archives of Disease in Childhood 2010;95(10):781–5.

44. Le Saux N, Howard A, Barrowman NJ, Gaboury I, Sampson M, Moher D. Shorter courses of parenteral antibiotic therapy do not appear to influence response rates for children with acute hematogenous osteomyelitis: a systematic review. BMC infectious diseases 2002;2(16).

For peer review only

Table 1: Baseline characteristics of children with *Staphylococcus aureus* bacteraemia included in the study

Baseline variable	Pre-IDC group N=28 patients (%)	IDC group N=35 patients (%)	Combined N=63 patients (%)
Male	20 (71.4)	20 (57.1)	40 (63.5)
Female	8 (28.6)	15 (42.9)	23 (36.5)
Median age in years (IQR)	4.3 (0.2 – 9.4)	3.4 (0.2 – 12.2)	3.4 (0.2 – 10.7)
Neonates	6 (20)	9 (25.0)	15 (23.8)
Prematurity	4 (66.7)	9 (100.0)	13 (86.7)
Median age in days (IQR)	11 (7 – 47)	26 (23 – 38)	25 (14 – 47)
Median birth weight in grams (IQR)	1117 (630-3535)	820 (755-1120)	830 (710 – 1330)
Congenital heart disease	5 (17.9)	8 (22.9)	13 (20.6)
Chronic Pulmonary Disease	3 (10.7)	5 (14.3)	8 (12.7)
Liver Disease	0	1 (2.9)	1 (1.6)
Malignancy	7 (25.0)	9 (25.7)	16 (25.4)
Metastatic cancer	2 (7.1)	1 (2.9)	3 (4.8)
Neurological condition	4 (14.3)	13 (37.1)	17 (27.0)
Diabetes mellitus	0	1 (2.9)	1 (1.6)
Skin Condition	3 (10.7)	1 (2.9)	4 (6.4)
Atopic dermatitis	3 (100.0)		3 (75.0)
Immunosuppression	6 (21.4)	8 (22.9)	14 (22.2)
Corticosteroid therapy	2	3	5
Anti-neoplastic	5	5	10
Neutropenia	2	0	2
	**N=30 episodes (%)**	**N=36 episodes (%)**	**N=66 episodes (%)**
Mode of Acquisition			
Community-acquired	11 (36.7)	10 (27.8)	21 (31.8)
Healthcare-associated	9 (30.0)	7 (19.4)	16 (24.2)
Hospital-acquired	10 (33.3)	19 (52.8)	29 (43.9)
Duration of symptoms of bacteremia (hours)			
0-24	21 (70.0)	18 (50.0)	39 (59.1)
25-72	1 (3.3)	6 (16.7)	7 (10.6)
>72	7 (23.3)	10 (27.8)	17 (25.8)
Unknown	1 (3.3)	2 (5.6)	3 (4.6)
Organism			
MSSA	28 (93.3)	33 (91.7)	61 (92.4)
MRSA	2 (6.7)	3 (8.3)	5 (7.6)
C-Reactive Protein (nmol/L)	333 (114 – 890)	448 (181 – 1081)	390 (133 –1005)
White cell count (10^9/L)	8.8 (5.8 – 15.6)	10.1 (6.9 – 18.4)	9.6 (6.3 – 16.5)
Neutrophils (x 10^9/L)	5.4 (3.6 – 9)	6.3 (4.9-11.8)	5.7 (3.6 – 11.8)
Platelets (x 10^9/L)	277 (151 – 374)	213 (101 – 270)	224 (140 – 301)

IDC = infectious diseases consultation; IQR = interquartile range; MSSA = methicillin-susceptible *Staphylococcus aureus*; MRSA = methicillin-resistant *Staphylococcus aureus*

For peer review only

Table 2: Clinical features of *Staphylococcus aureus* bacteraemia in children included in the study

Focus of infection at time of bacteremia	Pre-IDC group N=30 episodes (%)	IDC group N=36 episodes (%)	Combined N=66 episodes (%)	P-value
Unknown focus	7 (23.3)	2 (5.6)	9 (13.6)	NS
Intravascular catheter	8 (26.7)	22 (61.3)	30 (45.5)	0.013
Culture confirmed	5 (62.5)	11 (50.0)	16 (53.3)	NS
Thrombophlebitis	0	2 (5.7)	2 (3.0)	NS
Bone/Joint infection	8 (26.7)	8 (22.2)	16 (24.2)	NS<br>NS
Culture confirmed	4 (50.0)	2 (25.0)	6 (37.5)	
Lung	1 (3.3)	1 (2.8)	2 (3.0)	NS
Culture confirmed	0	1 (100.0)	1 (50)	NS
Skin & Soft tissue	7 (23.3)	9 (25.0)	16 (24.2)	NS
Culture confirmed	4 (57.1)	8 (88.9)	12 (75.0)	NS
Deep tissue abscess	2 (6.7)	1 (2.8)	3 (4.6)	NS<br>NS
Culture confirmed	2 (100.0)	0	2 (66.7)	
Other focus	2 (6.7)	4 (11.1)	6 (9.1)	NS
Defervescence at 72 hours				
Yes	18 (60.0)	20 (55.6)	38 (57.6)	NS
No	11 (36.7)	14 (38.9)	25 (37.9)	
Unknown	1 (3.3)	2 (5.6)	3 (4.6)	

IDC = infectious diseases consultation; NS = non-significant

For peer review only

Table 3: Risk factors for *Staphylococcus aureus* bacteraemia in children included in the study

Risk factor for SAB	Pre-IDC group N=30 episodes (%)	IDC group N=36 episodes (%)	All children N=66 (episodes)	P-value
Age <1 year	11 (36.7)	14 (38.9)	25 (37.9)	NS
Underlying medical condition	16 (53.3)	24 (66.7)	40 (60.6)	NS
Duration in hospital in days (IQR)<br>Prior to bacteremia*	11.5 (7.0-21.0)	19.0 (12.0-37.0)	16.0 (8.0-24.0)	NS
Prosthetic material	14 (46.7)	26 (72.2)	40 (60.6)	0.04
Intravascular line	13 (43.3)	23 (63.9)	36 (54.6)	NS
Endotracheal tube	0	4	4	NS
Other	1	6	8	NS
Corticosteroid therapy	2 (6.7)	3 (8.3)	5 (7.6)	NS
Surgery within previous 30 days	4 (13.3)	4 (11.1)	8 (12.1)	NS

IDC=infectious disease consultation; IQR=interquartile range; NS = non-significant; SAB=*Staphylococcus aureus* bacteremia
*Hospital-acquired infection only

For peer review only

Table 4: Comparison of the management and outcome of *Staphylococcus aureus* bacteraemia in children, pre- and post-introduction of an infectious disease consult service.

Quality indicator	Pre-IDC group N=30 episodes (%)	IDC group N=36 episodes (%)	P-Value
Median time to infectious diseases review in days (IQR)	N=4<br>3.5 (0.5-21.5)	N=34<br>2.0 (1.0-4.0)	NS
Repeat blood culture performed	26 (86.7)	32 (88.9)	NS
Time to repeat blood culture			
0-48 hours	20	18	NS
48-96 hours	8	13	NS
> 96 hours	1	1	NS
Repeat blood culture positive	6	6	NS
0-48 hours	3	4	NS
48-96 hours	0	0	NS
> 96 hours			
Echocardiogram performed			
Yes	10 (33.3)	29 (80.6)	0.0001
No	20 (66.7)	7 (19.4)	
Beta-Lactam therapy	27 (90.0)	34 (94.4)	NS
MSSA	25 (92.6)	33 (97.1)	
Removable focus of infection	7 (23.3)	22 (43.9)	0.003
Focus removed	6 (85.7)	21 (95.5)	NS
Median time to removal in days (IQR)	2.0 (2.0-2.0)	3.0 (1.0-18.0)	NS
Median time to appropriate antibiotics in days (IQR)	0.0 (0)	0.0 (0)	NS
Median duration of IV antibiotics in days (IQR)	13.5 (7.0-21.0)	18.0 (15.0-29.0)	0.035
Median duration of IV and/or oral antibiotics in days (IQR)	20.5 (16.0-42)	19.0 (15.0 – 29.5)	NS
**Complicated infection**	**N=15 episodes Days (IQR)**	**N=19 episodes Days (IQR)**	
Median duration of IV antibiotics	14.0 (6.0-21.0)	22 (15.0-39.0)	0.02
Median duration of IV or oral antibiotics	19.0 (17.0-43.0)	27.0 (16.0-39.0)	NS
Median duration of IV antibiotics if repeat blood culture positive	13.0 (6.0 – 14.0)	19.0 (15.0 – 27.0)	0.007
Met standard recommendation 28 days IV antibiotics (%)	2 (13.3)	8 (42.1)	NS
**Uncomplicated infection**	**N=15 episodes Days (IQR)**	**N=17 episodes Days (IQR)**	
Median duration of IV antibiotics	13.0 (7.0 – 22.0)	15.0 (14.0-21.0)	NS
Median duration of IV or oral antibiotics (IQR)	22.0 (14.0-32.0)	18.0 (14.0-29.0)	NS
Met standard			

For peer review only

recommendation of 14 days (%)	7 (46.7)	13 (68.4)	NS
**Outcomes**	**N=30 episodes Days (IQR)**	**N=36 episodes Days (IQR)**	
Median duration of hospital admission<br>Total<br>Complicated<br>Uncomplicated	 <br>14.0 (6.0-42.0)<br>20.0 (6.0 – 49.0)<br>7.0 (3.0-22.0)	 <br>16.5 (7.5-58)<br>25.0 (8.0-88.0)<br>11.0 (6.0-36.0)	 <br>NS<br>NS<br>NS
SAB recorded in discharge summary	24 (80.0)	29 (82.9)	NS
Secondary infection detected	1	3	NS
Outcomes 30-day post-SAB<br>Death<br>Recurrence	 0<br>0	 3<br>0	 NS<br>NS
Outcomes 30-90 days post SAB<br>Death<br>Recurrence	 2<br>2	 0<br>1	 NS<br>NS

IDC = infectious diseases consultation; IQR = Interquartile range; IV = intravenous; NS = non-significant; SAB = *Staphylococcus aureus* bacteremia; Mortality was analysed per patient (only the first episode was analysed).

For peer review only

Figure 1. Study schema of paediatric patients with *S. aureus* bacteraemia

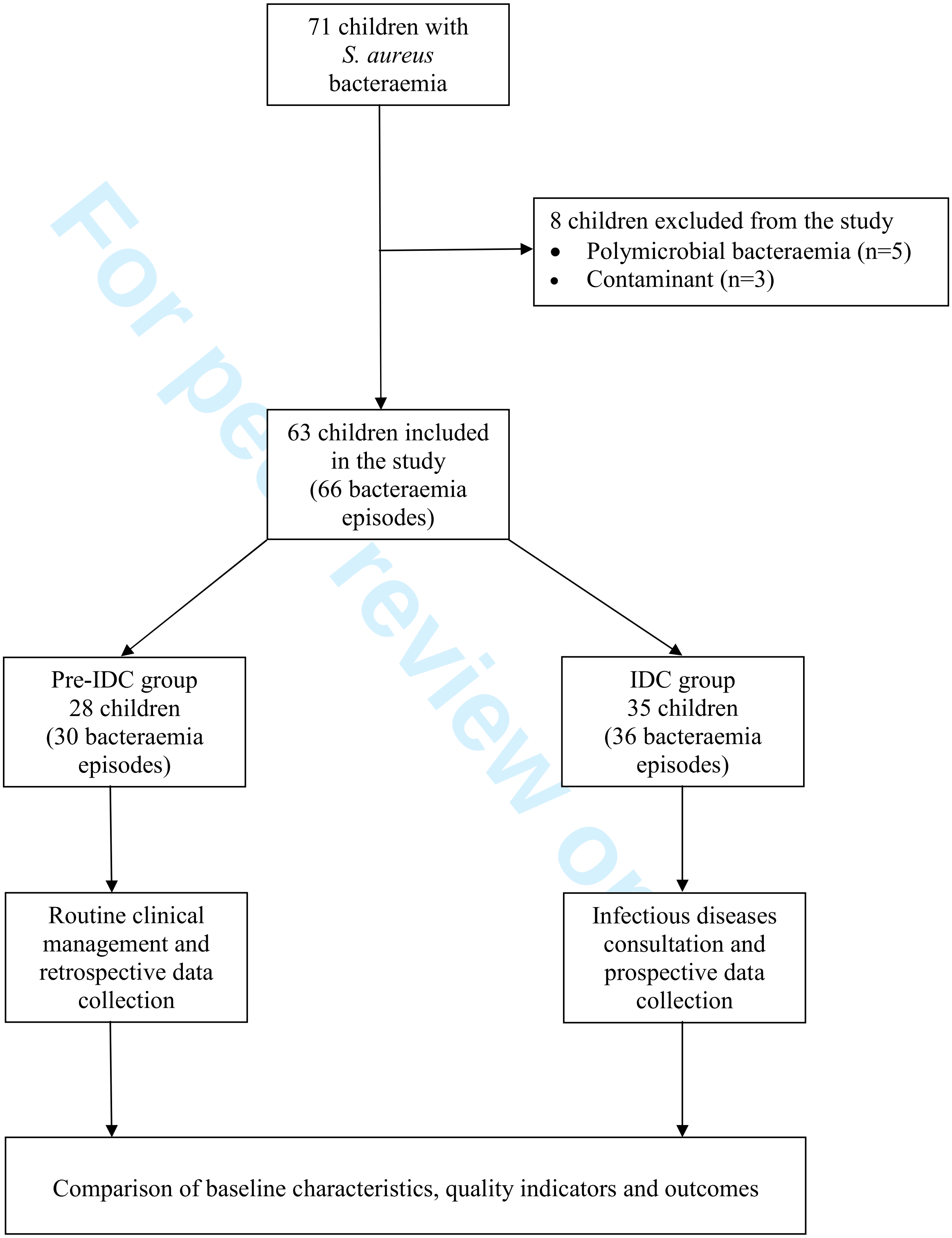

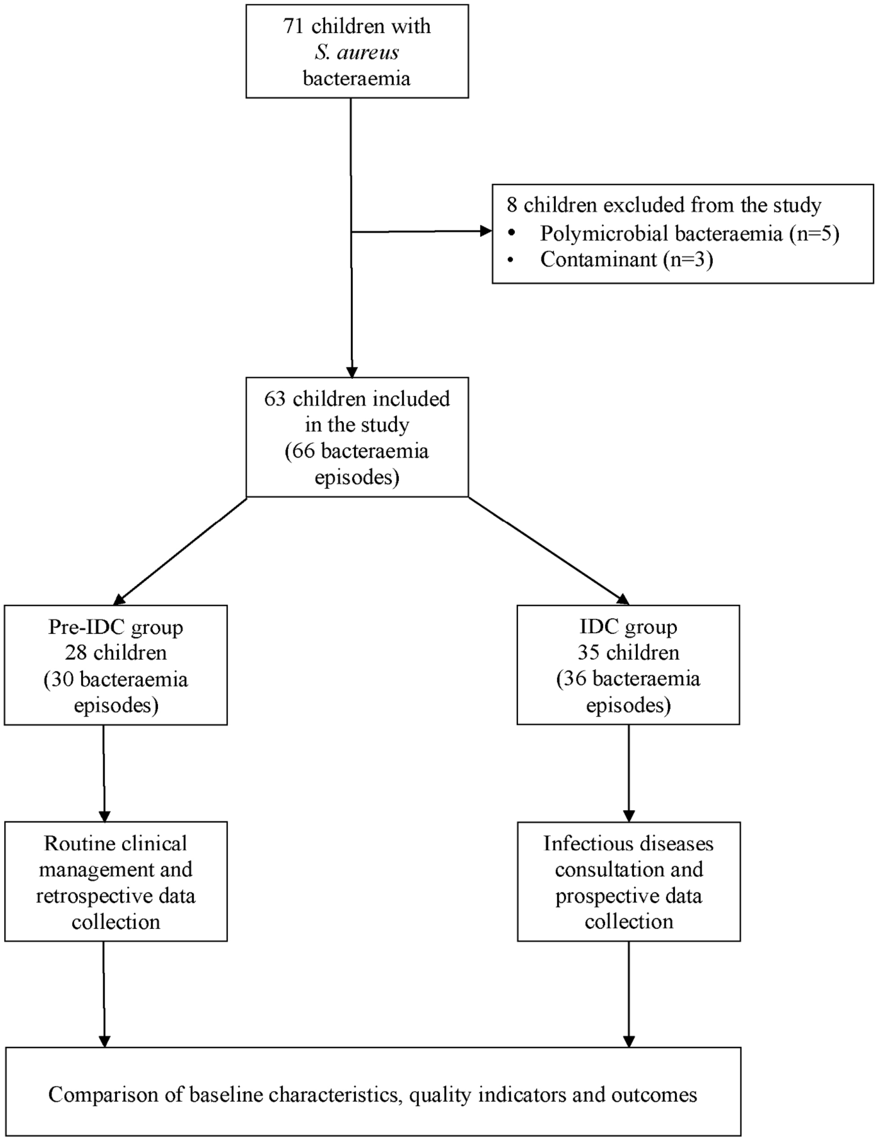

Figure 1. Study schema of paediatric patients with S. aureus bacteraemia
168x218mm (300 x 300 DPI)
